# Discrete maximal regularity of time-stepping schemes for fractional evolution equations

**DOI:** 10.1007/s00211-017-0904-8

**Published:** 2017-07-22

**Authors:** Bangti Jin, Buyang Li, Zhi Zhou

**Affiliations:** 1 Department of Computer Science, University College London, Gower Street, London, WC1E 6BT UK; 2 Department of Applied Mathematics, The Hong Kong Polytechnic University, Kowloon, Hong Kong

**Keywords:** 65M12, 65J10, 65M60, 34A08

## Abstract

In this work, we establish the maximal $\ell ^p$-regularity for several time stepping schemes for a fractional evolution model, which involves a fractional derivative of order $\alpha \in (0,2)$, $\alpha \ne 1$, in time. These schemes include convolution quadratures generated by backward Euler method and second-order backward difference formula, the L1 scheme, explicit Euler method and a fractional variant of the Crank–Nicolson method. The main tools for the analysis include operator-valued Fourier multiplier theorem due to Weis (Math Ann 319:735–758, 2001. doi:10.1007/PL00004457) and its discrete analogue due to Blunck (Stud Math 146:157–176, 2001. doi:10.4064/sm146-2-3). These results generalize the corresponding results for parabolic problems.

## Introduction

Maximal $L^p$-regularity is an important mathematical tool in studying the existence, uniqueness and regularity of solutions of nonlinear partial differential equations of parabolic type. A generator *A* of an analytic semigroup on a Banach space *X* is said to have maximal $L^p$-regularity, if the solution *u* of the following parabolic differential equation

1.1 $$\begin{aligned} \begin{aligned} u^\prime (t)&= A u + f\quad \forall t>0,\\ u(0)&=0, \end{aligned} \end{aligned}$$

satisfies the following estimate

1.2 $$\begin{aligned} \Vert u^\prime \Vert _{L^p(\mathbb {R}^+;X)} + \Vert Au\Vert _{L^p(\mathbb {R}^+;X)}\le c_{p,X}\Vert f\Vert _{L^p(\mathbb {R}^+;X)}\quad \forall f\in L^p(\mathbb {R}^+;X), \end{aligned}$$

with $1<p<\infty$. On a Hilbert space *X*, every generator of a bounded analytic semigroup has maximal $L^p$-regularity [45], and Hilbert spaces are only spaces for which this holds true [24]. Beyond Hilbert spaces, an important and very useful characterization of the maximal $L^p$-regularity was given by Weis [48] on UMD spaces in terms of the *R*-boundedness of a family of operators using the resolvent $R(z;A):=(z-A)^{-1}$; see Theorem 1 in Sect. 2 for details.

An important question from the perspective of numerical analysis is whether such maximal regularity estimates carry over to time-stepping schemes for discretizing the parabolic problem (1.1), which have important applications in numerical analysis of nonlinear parabolic problems [1, 2, 17, 29, 33]. This question has been studied in a number of works from different aspects [4, 5, 15, 16, 31, 32, 34]. Ashyralyev et al. [4] showed the following discrete maximal regularity: for all $f^n\in X,\,\, n=1,2,\dots$,

$$\begin{aligned} \tau ^{-1}\Vert (u^n-u^{n-1})_{n=1}^N\Vert _{\ell ^p(X)} + \Vert (Au^n)_{n=1}^N\Vert _{\ell ^p(X)}\le c_{p,X}\Vert (f^n)_{n=1}^N\Vert _{\ell ^p(X)} \end{aligned}$$

for the time-discrete solutions $u^n$, $n=1,2,\dots ,$ given by the implicit Euler method, where $\tau$ is the time step size and the constant $c_{p,X}$ is independent of $\tau$. A variant of the maximal $\ell ^p$-regularity for the Crank–Nicolson method was also shown in [4]. Recently, Kovács et al. [28] proved the discrete maximal regularity for the Crank–Nicolson, BDF and *A*-stable Runge–Kutta methods. Kemmochi and Saito [25, 26] proved the maximal $\ell ^p$-regularity for the $\theta$-method. In these works, the main tools are the maximal $L^p$-regularity characterization due to Weis [48] and its discrete analogue due to Blunck [10]. Independently, Leykekhman and Vexler [31] proved the maximal $L^p$-regularity of discontinuous Galerkin methods without using Blunck’s multiplier technique. The maximal $\ell ^p$-regularity of fully discrete numerical solutions have been investigated in [25, 26, 31] and [35] for parabolic equations with time-independent and time-dependent coefficients, respectively; also see [28, section 6].

The maximal $L^p$-regularity has also been studied for the following fractional evolution equation

1.3 $$\begin{aligned} \partial _t^\alpha u(t) = A u(t) + f\quad \forall t>0, \end{aligned}$$

together with the following initial condition(s)

$$\begin{aligned} \begin{aligned} u(0)&= 0,&\hbox {if} \quad 0<\alpha<1,\\ u(0)&= 0, \quad \partial _t u (0) = 0,&\hbox {if}\quad 1<\alpha <2. \end{aligned} \end{aligned}$$

In the model (1.3), the notation $\partial _t^\alpha u$ denotes the Caputo fractional derivative of order $\alpha$ of *u* with respect to time *t*, defined by [27, pp. 91]

$$\begin{aligned} \partial _t^\alpha u(t)= \frac{1}{\varGamma (n-\alpha )} \int _0^t(t-s)^{n -\alpha -1}\frac{d^n}{ds^n}u(s)ds, \quad n-1<\alpha <n, \, n\in \mathbb {N}, \end{aligned}$$

where the Gamma function $\varGamma (\cdot )$ is defined by $\varGamma (z)=\int _0^\infty s^{z-1}e^{-s}ds$, $\mathfrak {R}z>0$. With zero initial condition(s), it is identical with the Riemann–Liouville one [27, pp. 70]

$$\begin{aligned} ^R\partial _t^{\alpha } u(t)=\frac{1}{\varGamma (n-\alpha )} \frac{d^n}{dt^n}\int _0^t(t-s)^{n-\alpha -1}u(s)ds, \quad n-1<\alpha <n, \, n\in \mathbb {N}. \end{aligned}$$

Throughout, we use only the notation $\partial _t^\alpha u$ to denote either derivative. When $\alpha =1$, the fractional derivative $\partial _t^\alpha u(t)$ coincides with the usual first-order derivative $u^\prime (t)$, and accordingly, the fractional model (1.3) recovers the standard parabolic equation (1.1). In this paper we focus on the fractional cases $0< \alpha < 1$ and $1< \alpha < 2$, which are known as the subdiffusion and diffusion-wave equation, respectively. In analogy with Brownian motion for normal diffusion (1.1), the model (1.3) with $0<\alpha < 1$ is the macroscopic counterpart of continuous time random walk.

The fractional model (1.3) has received much attention in recent years, since it can adequately capture the dynamics of anomalous diffusion processes. For example, the subdiffusion equation, i.e., $\alpha \in (0,1)$, has been employed to describe transport in column experiments, thermal diffusion in media with fractal geometry, and flow in highly heterogeneous aquifers. See [40] for an extensive list of applications. The diffusion-wave equation, i.e., $\alpha \in (1,2)$, can be used to model mechanical wave propagation in viscoelastic media.

In a series of interesting works [6–8], Bazhalekov and collaborators have established the following maximal $L^p$-regularity for the fractional model (1.3): for any $1<p<\infty$, $u\in L^p(\mathbb {R}^+;D(A))$ and

1.4 $$\begin{aligned} \Vert \partial _t^\alpha u \Vert _{L^p(\mathbb {R}^+;X)} + \Vert Au\Vert _{L^p(\mathbb {R}^+;X)}\le c_{p,X}\Vert f\Vert _{L^p(\mathbb {R}^+;X)}\quad \forall f\in L^p(\mathbb {R}^+;X), \end{aligned}$$

under suitable conditions on the operator *A* (see Theorem 3 in Sect. 2 for details). Further, they applied the theory to analyze nonautonomous and semilinear problems [6, 8]. See also [44] for closely related maximal regularity results for Volterra integro-differential equations.

The discrete analogue of (1.4) is important for the numerical analysis of nonautonomous and nonlinear fractional evolution problems. The only existing result we are aware of is the very recent work of Lizama [37]. Specifically, Lizama studied the following fractional difference equation with $0<\alpha <1$:

$$\begin{aligned} \varDelta ^\alpha u^n=Tu^n+f^n, \end{aligned}$$

where $u^0=0$ and $\varDelta ^\alpha$ is a certain fractional difference operator. The author established the maximal $\ell ^p$-regularity for the problem, under the condition that the set $\{\delta (z)(\delta (z)-T)^{-1}:|z|=1,z\ne 1\}$ is *R*-bounded, with $\delta (z)=z^{1-\alpha }(1-z)^\alpha$, following the work of Blunck [10]. It can be interpreted as a time-stepping scheme: upon letting $T=\tau ^\alpha A$ and $f^n=\tau ^\alpha g^n$, we get $\tau ^{-\alpha } \varDelta ^\alpha u^n= Au^n+g^n$. Hence, it amounts to a convolution quadrature generated by the kernel $z^{1-\alpha }(1-z)^\alpha$. However, this scheme lacks the maximal $\ell ^p$-regularity if $A=\varDelta$, the Dirichlet Laplacian operator in bounded domains.

In this work, we address the following question: *Under which conditions do the time discretizations of* (1.3) *preserve the maximal* $\ell ^p$-*regularity, uniformly in the time step size* $\tau$? We provide an analysis for several time-stepping schemes, including the convolution quadratures generated by the implicit Euler method and second-order backward difference formula [11, 21], the L1 scheme [36, 46], the explicit Euler method [50] and a fractional variant of the Crank–Nicolson method. Amongst them, the convolution quadrature is relatively easy to analyze. In contrast, the L1 scheme and explicit Euler method are easy to implement, but challenging to analyze. The explicit Euler method requires a bounded numerical range of the operator *A* and the step size $\tau$ to be small enough. The maximal $\ell ^p$-regularity of the Crank–Nicolson method behaves like the implicit Euler scheme when $0<\alpha <1$ and like the explicit Euler scheme when $1<\alpha <2$. Our proof strategy follows closely the recent works [25, 28] and employs the (discrete) Fourier multiplier technique of Blunck [10].

The rest of the paper is organized as follows. In Sect. 2 we recall basic tools for showing maximal $\ell ^p$-regularity, including *R*-boundedness, UMD spaces, and Fourier multiplier theorems. Then four classes of time-stepping schemes, i.e., convolution quadrature, L1 scheme, explicit Euler method and a variant of the fractional Crank–Nicolson method, are discussed in Sects. 3–6, respectively. In Sect. 7, we discuss the extension to nonzero initial data. Last, in Sect. 8, we illustrate the results with several concrete examples.

We conclude the introduction with some notation. For a Banach space *X*, we denote by $\mathcal {B}(X)$ the set of all bounded linear operators from *X* into itself. For a linear operator *A* on *X*, we denote by $\sigma (A)$ and $\rho (A)$ its spectrum and resolvent set, respectively. We denote the unit circle in the complex plane $\mathbb {C}$ by $\mathbb {D}=\{z:|z|=1\}$, and $\mathbb {D}^\prime =\{z:|z|=1,z\ne \pm 1\}$. Given any $\theta \in (0,\pi ),$ the notation $\varSigma _\theta$ denotes the open sector $\varSigma _\theta :=\left\{ z\in \mathbb {C}: |\arg (z)|< \theta , z\ne 0\right\}$, where $\mathrm{arg}(z)$ denotes the argument of $z\in {\mathbb C}\backslash \{0\}$ in the range $(-\pi ,\pi ]$. Throughout, the notation *c* and *C*, with or without a subscript/superscript, denote a generic constant, which may differ at different occurrences, but it is always independent of the time step size $\tau$ and the number *N* of time steps.

## Preliminaries

In this section we collect basic results on the maximal $L^p$-regularity and related concepts, especially *R*-boundedness, UMD spaces, and Fourier multiplier theorems, used in the fundamental work of Weis [48], where he characterized the maximal $L^p$-regularity of an operator *A* in terms of its resolvent operator $R(z;A):=(z-A)^{-1}$. We refer readers to the review [30] for details.

### *R*-boundedness

The concept of *R*-boundedness plays a crucial role in Weis’ operator-valued Fourier multiplier theorem and its discrete analogue. A collection of operators $\mathcal {M}=\{M(\lambda ): \lambda \in \Lambda \}\subset \mathcal {B}(X)$ is said to be *R*-bounded if there is a constant $c>0$ such that any finite subcollection of operators $\{M(\lambda _j)\}_{j=1}^l$ satisfies

2.1 $$\begin{aligned} \int _0^1\bigg \Vert \sum _{j=1}^lr_j(s)M(\lambda _j)v_j\bigg \Vert _Xds\le c\int _0^1\bigg \Vert \sum _{j=1}^lr_j(s)v_j\bigg \Vert _Xds\quad \forall v_1,v_2,\ldots ,v_l\in X, \end{aligned}$$

where $r_j(s)=\mathrm {sign}\sin (2j\pi s)$, $j=1,2,\ldots ,$ are the Rademacher functions defined on the interval [0, 1]. The infimum of the constant *c* satisfying (2.1), denoted by $R(\mathcal {M})$ below, is called the *R*-bound of the set $\mathcal {M}$. In particular, if $\Lambda \subset \{z\in \mathbb {C}: |z|\le c_0\}$ for some $c_0>0$, then the set $\{\lambda I: \lambda \in \Lambda \}$ is *R*-bounded with an *R*-bound $2c_0$. This fact will be used extensively below.

There are a number of basic properties of *R*-bounded sets, summarized below. They follow from definition and the proofs can be found in [30].

#### Lemma 1

Let $\mathcal {T}\subset \mathcal {B}(X)$ be an *R*-bounded set. Then the following statements hold.

(i) If $\mathcal {S}\subset \mathcal {T}$, then $\mathcal {S}$ is *R*-bounded with $R(\mathcal {S})\le R(\mathcal {T})$.
(ii) The closure $\overline{\mathcal {T}}$ in $\mathcal {B}(X)$ is also *R*-bounded, and $R(\overline{\mathcal {T}})=R(\mathcal{T})$.
(iii) If $\mathcal {S}\subset \mathcal {B}(X)$ is *R*-bounded, then the union $\mathcal{S}\cup \mathcal{T}$ and sum $\mathcal{S+T}$ are *R*-bounded, with $R(\mathcal{S\cup T})\le R(\mathcal{S})+R(\mathcal{T})$ and $R(\mathcal{S+T})\le R(\mathcal{S})+R(\mathcal{T})$.
(iv) If $\mathcal{S}\subset \mathcal {B}(X)$ is *R*-bounded, then $\mathcal{ST}$ is *R*-bounded with $R(\mathcal{ST})=R(\mathcal{S})R(\mathcal{T})$.
(v) The convex hull $\mathrm {CH}(\mathcal{T})$ is *R*-bounded with $R(\mathrm {CH}(\mathcal{T}))\le R(\mathcal{T})$.
(vi) The absolutely convex hull of $\mathcal{T}$, denoted by $\mathrm {ACH}_\mathbb {C}(\mathcal{T})$, is *R*-bounded, with $R(\mathrm {ACH}_\mathbb {C}(\mathcal{T}))\le 2R(\mathcal{T})$.

The following useful result is a slight extension of [10, Corollary 3.5].

#### Lemma 2

Let *A* be a closed and densely defined operator in *X*, and $\delta \in (0,\pi )$. If $\{zR(z;A):z\in \varSigma _{\delta }\}$ is *R*-bounded, then there exists $\delta ^\prime \in (\delta ,\pi )$ such that $\{zR(z;A): z\in \varSigma _{\delta ^\prime }\}$ is *R*-bounded.

#### Proof

In fact, the *R*-boundedness of $\{zR(z;A):z\in \varSigma _{\delta }\}$ implies the *R*-boundedness of $\{\rho e^{\mathrm {i}\delta } R(\rho e^{\mathrm {i}\delta };A):\rho >0\}$. Via a rotation in the complex plane $\mathbb {C}$, we see that $\{\rho \mathrm { i} R(\rho \mathrm {i} ;e^{\mathrm{i}(\pi /2-\delta )}A): \rho >0\}$ is *R*-bounded. Then the proof of [10, Corollary 3.5] implies the *R*-boundedness of $\{w R(w;e^{\mathrm{i}(\pi /2-\delta )}A): \pi /2<\arg (w)<\pi /2+\vartheta \}$ for some small $\vartheta >0$. By rotating back in the complex plane $\mathbb {C}$, we have the *R*-boundedness of $\{z R(z;A): \delta<\arg (z)<\delta +\vartheta \}$. The *R*-boundedness of $\{z R(z;A): -\delta -\vartheta<\arg (z)<-\delta \}$ follows similarly. Overall, the set $\{z R(z;A): z\in \varSigma _{\delta +\vartheta }\}$ is *R*-bounded. $\square$

### Operator-valued multiplier theorems on UMD spaces

Now we recall the concept of UMD spaces, which is essential for multiplier theorems. Let $S(\mathbb {R};X)$ denote the space of rapidly decreasing *X*-valued functions. A Banach space *X* is said to be a UMD space if the Hilbert transform

$$\begin{aligned} Hf(t)=\mathrm {P.V.}\int _\mathbb {R}\frac{1}{t-s}f(s)ds, \end{aligned}$$

defined on the space $S(\mathbb {R};X)$, can be extended to a bounded operator on $L^p(\mathbb {R};X)$ for all $1<p<\infty$. Equivalently, this can be characterized by unconditional martingale differences, hence the abbreviation UMD. Examples of UMD spaces include Hilbert spaces, finite-dimensional Banach spaces, and $L^q(\varOmega ,d\mu )$ ($(\varOmega ,\mu )$ is a $\sigma$-finite measure space, $1<q<\infty$), and its closed subspaces (e.g., Sobolev spaces $W^{m,p}(\varOmega )$, $1<p<\infty$), and the product space of UMD spaces. Throughout, *X* always denotes a UMD space. Next we recall the concept of *R*-sectoriality operator. The definition below is equivalent to [30, Section 1.11] by changing *A* to $-A$ and changing $\theta$ to $\pi -\theta$.

#### Definition 1

An operator $A:D(A)\rightarrow X$ is said to be sectorial of angle $\theta$ if the following three conditions are satisfied:

(i) $A:D(A)\rightarrow X$ is a closed operator and its domain *D*(*A*) is dense in *X*;
(ii) The spectrum of *A* is contained in the sector ${\mathbb C}\backslash \varSigma _{\theta }$;
(iii) The set of operators $\{zR(z;A) :z\in \varSigma _{\theta }\}$ is bounded in $\mathcal {B}(X)$.

Similarly, *A* is said to be *R*-sectorial of angle $\theta$ if (i), (ii) and the following condition hold:

(iii$$^\prime $$)
The set of operators $\{zR(z;A) :z\in \varSigma _{\theta }\}$ is *R*-bounded in $\mathcal {B}(X)$.

The following theorem is a simple consequence of Dore [13, Theorem 2.1] and Weis [48, Theorem 4.2].

#### Theorem 1

A densely defined closed operator *A* on a UMD space *X* has maximal parabolic $L^p$-regularity (1.2) if and only if *A* is *R*-sectorial of angle $\pi /2$.

The “if” direction in Theorem 1 is a consequence of the following operator-valued Fourier multiplier theorem [48, Theorem 3.4], where $\mathcal {F}$ denotes the Fourier transform on $\mathbb {R}$, i.e.,

$$\begin{aligned} \mathcal {F}f(\xi ) = \int _\mathbb {R}e^{-\mathrm {i}\xi t}f(t)dt\quad \xi \in \mathbb {R}. \end{aligned}$$

#### Theorem 2

Let *X* be a UMD space. Let $M:\mathbb {R}{\setminus }\{0\}\rightarrow \mathcal {B}(X)$ be differentiable such that the set

$$\begin{aligned} \{M(\xi ):\xi \in \mathbb {R}{\setminus }\{0\}\}\cup \{\xi M^\prime (\xi ):\xi \in \mathbb {R}{\setminus }\{0\}\}\hbox { is } R\hbox {-bounded}, \end{aligned}$$

with an *R*-bound $c_R$. Then $\mathcal {M}f:=\mathcal {F}^{-1}(M(\cdot )(\mathcal {F}f)(\cdot ))$ extends to a bounded operator

$$\begin{aligned} \mathcal {M}: L^p(\mathbb {R},X)\rightarrow L^p(\mathbb {R},X)\quad \hbox {for }\; 1<p<\infty . \end{aligned}$$

Further, there exists $c_{p,X}>0$ independent of *M* such that the operator norm of $\mathcal {M}$ is bounded by $c_Rc_{p,X}$.

Using Theorem 2, one can similarly show the following maximal regularity result for the fractional model (1.3) [6–8], which naturally extends the “if” part of Theorem 1 to the fractional case.

#### Theorem 3

Let *A* be an *R*-sectorial operator of angle $\alpha \pi /2$ on a UMD space *X*. Then the solution of (1.3) satisfies the maximal $L^p$-regularity estimate (1.4) for any $1<p<\infty$.

In this work, we discuss the discrete analogue of Theorem 3 for a number of time-stepping schemes for solving (1.3), under the same condition on the operator *A*, using a discrete version of Theorem 2 due to Blunck [10]. We slightly abuse $\mathcal {F}$ for the Fourier transform on $\mathbb {Z}_+:=\{n\in {\mathbb Z}: n\ge 0\}$, which maps a sequence $(f^n)_{n=0}^\infty$ to its Fourier series on the interval $(0,2\pi )$, i.e.,

$$\begin{aligned} \mathcal {F}f(\theta ) = \sum _{n=0}^\infty e^{-\mathrm {i}n\theta }f^n, \quad \forall \, \theta \in (0,2\pi ), \end{aligned}$$

and let ${\mathcal F}_\theta ^{-1}$ denote the inverse Fourier transform with respect to $\theta$, i.e.,

$$\begin{aligned} {\mathcal F}_\theta ^{-1}f(\theta ) = \Big (\frac{1}{2\pi }\int _0^{2\pi } f(\theta )e^{\mathrm {i} n\theta } d\theta \Big )_{n=0}^\infty . \end{aligned}$$

The following result is an immediate consequence of [10, Theorem 1.3], and will be used extensively; see also [25] for a proof with a more explicit constant. The statement is equivalent to Blunck’s original theorem via the transformation $\xi =e^{-\mathrm{i}\theta }$, but avoids introducing a different notation $\widetilde{M}(\theta )$.

#### Theorem 4

Let *X* be a UMD space, and let $M:{\mathbb D}'\rightarrow \mathcal {B}(X)$ be differentiable such that the set

2.2 $$\begin{aligned} \left\{ M(\xi ):\xi \in {\mathbb D}'\right\} \cup \left\{ (1-\xi ) (1+\xi ) M^\prime (\xi ): \xi \in {\mathbb D}'\right\} \end{aligned}$$

is *R*-bounded, with an *R*-bound $c_R$. Then $\mathcal {M}f:=\mathcal {F}_\theta ^{-1}( M(e^{-\mathrm {i} \theta }) (\mathcal {F}f)(\theta ))$ extends to a bounded operator

$$\begin{aligned} \mathcal {M}:\ell ^p(\mathbb {Z}_+,X)\rightarrow \ell ^p(\mathbb {Z}_+,X)\quad \hbox {for } \,1<p<\infty . \end{aligned}$$

Further, there exists a $c_{p,X}>0$ independent of *M* such that the operator norm of $\mathcal {M}$ is bounded by $c_Rc_{p,X}$.

To simplify the notations, for a given sequence $\{M_n\}_{n=0}^\infty$ of operators on a UMD space *X*, we define the generating function

2.3 $$\begin{aligned} M(\xi ): = \sum _{n=0}^\infty M_n\xi ^n\quad \forall \xi \in {\mathbb D}' . \end{aligned}$$

Likewise, the generating function $f(\xi )$ of a sequence $(f^n)_{n=0}^\infty$ is defined by

2.4 $$\begin{aligned} f(\xi ):=\sum _{n=0}^\infty f^n\xi ^n. \end{aligned}$$

The operator $\mathcal {M}$ is then given by $(\mathcal {M}f)_n=\sum _{j=0}^nM_{n-j}f^j$, $n=0,1,\ldots$. The generating function satisfies the convolution rule

2.5 $$\begin{aligned} (f*g)(\xi )=f(\xi )g(\xi ), \end{aligned}$$

where $$(f*g)_n:=\sum _{j=0}^n f^j g^{n-j},$$ $$n= 0,1,\ldots $$.

## Convolution quadrature

The convolution quadrature of Lubich (see the review [38] and references therein) presents one versatile framework for developing time-stepping schemes for the model (1.3). One salient feature is that it inherits excellent stability property (of that for ODEs). We shall consider convolution quadrature generated by backward Euler (BE) and second-order backward difference formula (BDF2), whose error analysis has been carried out in [11, 21].

### BE scheme

We first illustrate basic ideas to prove discrete maximal regularity on BE scheme in time *t*, with a constant time step size $\tau >0$. The BE scheme for (1.3) is given by: given $u^0=0$, find $u^n\in X$

3.1 $$\begin{aligned} \bar{\partial }_\tau ^\alpha u^n = A u^n + f^n,\quad n=1,2,\ldots \end{aligned}$$

where the BE approximation $\bar{\partial }_\tau ^\alpha u^n$ to $\partial _t^\alpha u(t_n)$ is given by

3.2 $$\begin{aligned} \bar{\partial }_\tau ^\alpha u^n = \tau ^{-\alpha }\sum _{j=0}^nb_{n-j}u^j\quad \hbox {with } \sum _{j=0}^\infty b_j\xi ^j=\delta (\xi )^\alpha :=(1-\xi )^\alpha , \end{aligned}$$

where $\delta (\xi )=1-\xi$ is the characteristic function of the BE method.

Now we can state the discrete maximal regularity of the BE scheme (3.1).

#### Theorem 5

Let *X* be a UMD space, $0<\alpha <1$ or $1<\alpha <2$, and let *A* be an *R*-sectorial operator on *X* of angle $\alpha \pi /2$. Then the BE scheme (3.1) has the following maximal $\ell ^p$-regularity

$$\begin{aligned} \Vert (\bar{\partial }_\tau ^\alpha u^n)_{n=1}^N\Vert _{\ell ^p(X)}+ \Vert (Au^n)_{n=1}^N\Vert _{\ell ^p(X)}\le c_{p,X}c_R\Vert (f^n)_{n=1}^N\Vert _{\ell ^p(X)}, \end{aligned}$$

where the constant $c_{p,X}$ is independent of *N*, $\tau$ and *A*, and $c_R$ denotes the *R*-bound of the set of operators $\{zR(z;A) :z\in \varSigma _{\alpha \pi /2}\}$.

#### Proof

By multiplying both sides of (3.1) by $\xi ^n$ and summing over *n*, we have

$$\begin{aligned} \sum _{n=1}^\infty \xi ^n \bar{\partial }_\tau ^\alpha u^n - \sum _{n=1}^\infty Au^n\xi ^n = \sum _{n=1}^\infty f^n\xi ^n. \end{aligned}$$

It suffices to compute the term $\sum _{n=1}^\infty \xi ^n \bar{\partial }_\tau ^\alpha u^n$. By noting $u^0=0$, the definition of the BE approximation (3.2) and discrete convolution rule (2.5), we deduce

$$\begin{aligned} \begin{aligned} \sum _{n=1}^\infty \xi ^n \bar{\partial }_\tau ^\alpha u^n&= \tau ^{-\alpha }\sum _{n=0}^\infty \xi ^n \sum _{j=0}^nb_{n-j}u^j = \tau ^{-\alpha }\left( \sum _{n=0}^\infty u^n\xi ^n\right) \left( \sum _{n=0}^\infty b_n\xi ^n\right) \\&=\tau ^{-\alpha }\delta (\xi )^\alpha u(\xi ). \end{aligned} \end{aligned}$$

Consequently, upon letting $f^0=0$, we arrive at

$$\begin{aligned} (\tau ^{-\alpha }\delta _\tau (\xi )^\alpha -A) u(\xi ) = f(\xi ). \end{aligned}$$

Since $\tau ^{-1}\delta (\xi )\in \varSigma _{\pi /2}$ for $\xi \in {\mathbb D}'$, we have $\tau ^{-\alpha }\delta (\xi )^\alpha \in \varSigma _{ \alpha \pi /2}$ for $\xi \in {\mathbb D}'$. The *R*-sectoriality of angle $\alpha \pi /2$ of the operator *A* ensures that the operator $\tau ^{-\alpha } \delta (\xi )^\alpha -A$ is invertible. Meanwhile, the generating function of the BE approximation $\bar{\partial }_\tau ^\alpha u$ is given by

$$\begin{aligned} (\bar{\partial }_\tau ^\alpha u)(\xi )&= \sum _{n=0}^\infty \xi ^n\bar{\partial }_\tau ^\alpha u^n =\tau ^{-\alpha }\delta (\xi )^\alpha u(\xi ) = M(\xi )f(\xi ). \end{aligned}$$

with $M(\xi ) =\tau ^{-\alpha }\delta (\xi )^\alpha (\tau ^{-\alpha }\delta (\xi )^\alpha -A)^{-1}$. Appealing to the *R*-sectoriality of *A* again gives that *zR*(*z*; *A*) is analytic and *R*-bounded in the sector $\varSigma _{\alpha \pi /2}$, which imply that $M(\xi )$ is differentiable and *R*-bounded for $\xi \in {\mathbb D}'$. Direct computation yields

$$\begin{aligned} (1-\xi )M^\prime (\xi ) = - \alpha M(\xi ) + \alpha M(\xi )^2, \end{aligned}$$

which together with Lemma 1 (iii)–(iv) implies the *R*-boundedness of the set (2.2). Then the desired result follows from Theorem 4. $\square$

#### Remark 1

The BE scheme (3.2) is identical with the Grünwald–Letnikov formula, a popular difference analogue of the Riemann–Liouville fractional derivative $\partial _t^\alpha u$ [43], which has been customarily employed for discretizing (1.3).

### Second-order BDF scheme

Next we consider the convolution quadrature generated by the second-order backward difference formula (BDF2) for discretizing the model (1.3):

3.3 $$\begin{aligned} \bar{\partial }_\tau ^\alpha u^n = Au^n + f^n,\quad n\ge 2 \end{aligned}$$

where the BDF2 approximation $\bar{\partial }_\tau ^\alpha u^n$ to $\partial _t^\alpha u(t_n)$, $t_n=n\tau$, is given by

3.4 $$\begin{aligned} \bar{\partial }_\tau ^\alpha u^n = \tau ^{-\alpha }\sum _{j=0}^n b_{n-j}u^{j}\quad \hbox {with } \sum _{j=0}^\infty b_j\xi ^j=\delta (\xi )^\alpha , \end{aligned}$$

with the characteristic function $\delta (\xi )$

3.5 $$\begin{aligned} \delta (\xi ) = \tfrac{3}{2}-2\xi +\tfrac{1}{2}\xi ^2. \end{aligned}$$

We approximate the fractional derivative $\partial _t^\alpha u(t_n)$ by the BDF2 convolution quadrature (3.4), and consider the scheme (3.3) with zero starting values $u^0=u^1=0$. Note that for the BDF2 scheme (and other higher-order linear multistep methods), the initial steps have to be corrected properly in order to achieve the desired accuracy [11, 21]. The next result gives the discrete maximal regularity of the scheme (3.3).

#### Theorem 6

Let *X* be a UMD space, $0<\alpha <1$ or $1<\alpha <2$, and let *A* be an *R*-sectorial operator on *X* of angle $\alpha \pi /2$. Then the BDF2 scheme (3.3) satisfies the following discrete maximal regularity

$$\begin{aligned} \Vert (\bar{\partial }_\tau ^\alpha u^n)_{n=2}^N\Vert _{\ell ^p(X)} +\Vert (Au^n)_{n=2}^N\Vert _{\ell ^p(X)}\le c_{p,X}c_R\Vert (f_n)_{n=2}^N\Vert _{\ell ^p(X)}, \end{aligned}$$

where the constant $c_{p,X}$ is independent of *N*, $\tau$ and *A*, and $c_R$ denotes the *R*-bound of the set of operators $\{zR(z;A) :z\in \varSigma _{\alpha \pi /2}\}$.

#### Proof

In a straightforward manner, upon letting $f^0=f^1=0$, we obtain

$$\begin{aligned} (\tau ^{-\alpha }\delta (\xi )^\alpha -A)u(\xi ) = f(\xi ), \end{aligned}$$

where $\delta (\xi )$ is defined in (3.5). Since BDF2 is *A*-stable (for ODEs), i.e., $\mathfrak {R}\delta (\xi )> 0$ for $\xi \in \mathbb {D}^\prime$, we have $\tau ^{-\alpha }\delta (\xi )^\alpha \subset \varSigma _{\alpha \pi /2}$. This and the *R*-sectoriality (of angle $\alpha \pi /2$) of the operator *A* implies that $\tau ^{-\alpha }\delta (\xi )^\alpha -A$ is invertible for $\xi \in \mathbb {D}'$. Further, direct computation gives

$$\begin{aligned} (\bar{\partial }_\tau ^\alpha u)(\xi )=M(\xi )f(\xi )\quad \hbox {with } M(\xi ) = \tau ^{-\alpha }\delta (\xi )^\alpha (\tau ^{-\alpha }\delta (\xi )^\alpha -A)^{-1}. \end{aligned}$$

The *R*-sectoriality of the operator *A* implies the *R*-boundedness of the set $\{M(\xi ): \xi \in \mathbb {D}^\prime \}$. Meanwhile,

$$\begin{aligned} (1-\xi )M^\prime (\xi ) = d(\xi ) M(\xi ) - d(\xi ) M(\xi )^2,\quad \hbox {with }d(\xi )=\alpha \frac{2(\xi -2)}{3-\xi }. \end{aligned}$$

Since $d(\xi )$ is bounded on $\mathbb {D}'$, Lemma 1 (iii)–(iv) and Theorem 4 give the desired assertion. $\square$

## L1 scheme

Now we discuss one time-stepping scheme of finite difference type for simulating subdiffusion—the L1 scheme [36, 46]—which is easy to implement and converges robustly for nonsmooth data, hence very popular. However, unlike convolution quadrature, finite difference type methods are generally challenging to analyze. For the subdiffusion case, i.e., $\alpha \in (0,1)$, it approximates the (Caputo) fractional derivative $\partial _t^\alpha u(t_n)$ with a time step size $\tau$ by

4.1 $$\begin{aligned} \partial _t^\alpha u(t_n)= & {} \frac{1}{\varGamma (1-\alpha )}\sum ^{n-1}_{j=0}\int ^{t_{j+1}}_{t_j} u^\prime (s)(t_n-s)^{-\alpha }\, ds \nonumber \\\approx & {} \frac{1}{\varGamma (1-\alpha )}\sum ^{n-1}_{j=0} \frac{u(t_{j+1})-u(t_j)}{\tau }\int _{t_j}^{t_{j+1}}(t_n-s)^{-\alpha }ds \nonumber \\= & {} \sum _{j=0}^{n-1}b_j\frac{u(t_{n-j})-u(t_{n-j-1})}{\tau ^\alpha }\nonumber \\= & {} \tau ^{-\alpha } [b_0u(t_n)-b_{n-1}u(t_0)+\sum _{j=1}^{n-1}(b_j-b_{j-1})u(t_{n-j})] =:\bar{\partial }_\tau ^\alpha u^n.\qquad \quad \end{aligned}$$

where the weights $b_j$ are given by

4.2 $$\begin{aligned} b_j=((j+1)^{1-\alpha }-j^{1-\alpha })/\varGamma (2-\alpha ),\ j=0,1,\ldots ,N-1. \end{aligned}$$

For the case $\alpha \in (1,2)$, the L1 scheme reads [46]

$$\begin{aligned} \partial _t^\alpha u(t_{n-\frac{1}{2}})&\approx \frac{\tau ^{-\alpha }}{\varGamma (3-\alpha )} \Big [a_0\delta _t u^{n-\frac{1}{2}} - \sum _{j=1}^{n-1}(a_{n-j-1}-a_{n-j})\delta _t u^{j-\frac{1}{2}}-a_{n-1}\tau \partial _tu(0) \Big ] \\&=:\bar{\partial }_\tau ^\alpha u^{n}, \end{aligned}$$

where $\delta _tu^{j-\frac{1}{2}}=u^{j}-u^{j-1}$ denotes central difference, and $a_j=(j+1)^{2-\alpha }-j^{2-\alpha }$, and we have abused the notation $\bar{\partial }_\tau ^\alpha u^n$ for approximating $\partial _t^\alpha u(t_{n-\frac{1}{2}})$. Formally, it can be obtained by applying (4.1) to the first derivative $\partial _t u$, in view of the identity $\partial _t^\alpha u = \partial _t^{\alpha -1} (\partial _t u)$, and then discretizing the $\partial _tu$ with the Crank–Nicolson type method. The scheme requires $\partial _tu(0)$, in addition to the initial condition *u*(0). Accordingly, we approximate the right hand side of (1.3) by a Crank–Nicolson type scheme. In sum, the L1 scheme reads

4.3 $$\begin{aligned} \left\{ \begin{aligned} \bar{\partial }_\tau ^\alpha u^n&= Au^n + f^n,&0<\alpha<1,\\ \bar{\partial }_\tau ^{\alpha }u^{n}&= A(u^n+u^{n-1})/2 + (f^n+f^{n-1})/2,&1<\alpha <2. \end{aligned}\right. \end{aligned}$$

### Remark 2

For $\alpha \in (0,1)$, Lin and Xu [36] proved that the L1 scheme is uniformly $O(\tau ^{2-\alpha })$ accurate for $C^2$ solutions; and for $\alpha \in (1,2)$, Sun and Wu [46] showed that it is uniformly $O(\tau ^{3-\alpha })$ accurate for $C^3$ solutions. It is worth noting that even for smooth initial data and source term, the solution of fractional-order PDEs may not be $C^2$ in general. In fact, the L1 scheme is generally only first-order [20, 23].

For the analysis, we recall the polylogarithmic function $\mathrm {Li}_p(z)$, $p\in \mathbb {R}$ and $z\in \mathbb {C}$, defined by

$$\begin{aligned} \mathrm {Li}_p(z)=\sum _{j=1}^\infty \frac{z^j}{j^p}. \end{aligned}$$

The function $\mathrm {Li}_p(z)$ is well defined on $\{z:|z|<1\}$, and it can be analytically continued to the split complex plane $\mathbb {C}{\setminus } [1,\infty )$ [14]. With $z=1$, it recovers the Riemann zeta function $\zeta (p)=\mathrm {Li}_p(1)$. First we state the solution representation.

### Lemma 3

The discrete solution $u(\xi )$ of the L1 scheme (4.3) satisfies

4.4 $$\begin{aligned} (\tau ^{-\alpha }\delta (\xi )-A)u(\xi )= f(\xi ), \end{aligned}$$

with the generating functions

$$\begin{aligned}&\delta (\xi )= \left\{ \begin{array}{ll}\displaystyle \frac{(1-\xi )^2}{\xi \varGamma (2-\alpha )}\mathrm {Li}_{\alpha -1}(\xi ), &{}\quad \alpha \in (0,1),\\ \displaystyle \frac{2(1-\xi )^3}{\xi (1+\xi )\varGamma (3-\alpha )}\mathrm {Li}_{\alpha -2}(\xi ), &{}\quad \alpha \in (1,2), \end{array}\right. \\&f(\xi )= \left\{ \begin{array}{l} \displaystyle \sum _{n=1}^\infty f^n\xi ^n,\quad \alpha \in (0,1),\\ \displaystyle \frac{\xi }{1+\xi }\sum _{n=0}^\infty f^n\xi ^n + \frac{1}{1+\xi }\sum _{n=1}^\infty f^n\xi ^n, \quad \alpha \in (1,2). \end{array}\right. \end{aligned}$$

### Proof

We first show the representation for $\alpha \in (0,1)$, and the case $\alpha \in (1,2)$ is analogous. Multiplying both sides of (4.3) by $\xi ^n$ and summing over *n* yield

$$\begin{aligned} \sum _{n=1}^\infty \bar{\partial }_\tau ^\alpha u^n\xi ^n - A u(\xi ) = \sum _{n=1}^\infty f^n\xi ^n, \end{aligned}$$

upon noting $u^0=0$. Now we focus on the term $\sum _{n=1}^\infty \bar{\partial }_\tau ^\alpha u^n\xi ^n$. Since $u^0=0$, by the convolution rule (2.5), we have

$$\begin{aligned} \begin{aligned} \sum _{n=1}^\infty \bar{\partial }_\tau ^\alpha u^n\xi ^n&= \tau ^{-\alpha }\sum _{n=1}^\infty \Big (b_0u^n+\sum _{j=1}^{n-1}(b_j-b_{j-1})u^{n-j}\Big ) \xi ^n\\&= \tau ^{-\alpha } \sum _{n=1}^\infty \Big (\sum _{j=0}^{n-1} b_j u^{n-j}\Big )\xi ^n - \tau ^{-\alpha }\sum _{n=1}^\infty \Big (\sum _{j=1}^{n-1} b_{j-1} u^{n-j}\Big )\xi ^n \\&= \tau ^{-\alpha }(1-\xi ) b(\xi ) u (\xi ). \end{aligned} \end{aligned}$$

Using the polylogarithmic function $\mathrm {Li}_p(z)$, $b(\xi )$ is given by

$$\begin{aligned} b(\xi )&= \frac{1}{\varGamma (2-\alpha )}\sum _{{j=0}}^\infty (( j+1 )^{1-\alpha }-j^{1-\alpha }) \xi ^j\\&= \frac{1-\xi }{\xi \varGamma (2-\alpha )} \sum _{j=1}^\infty j^{1-\alpha } \xi ^j= \frac{(1-\xi )\mathrm {Li}_{\alpha -1}(\xi )}{\xi \varGamma (2-\alpha )}, \end{aligned}$$

from which the desired solution representation follows directly. $\square$

We shall need the following result, which is of independent interest.

### Lemma 4

For $\alpha \in (0,1)$ and $\xi \in \mathbb {D}'$, we have $\psi (\xi ) := \frac{(1-\xi )^2}{\xi }\mathrm {Li}_{\alpha -1}(\xi )\in \varSigma _{\frac{\pi \alpha }{2}}$.

### Proof

It suffice to consider $\xi =e^{-\mathrm {i}\theta }$ with $\theta \in (0,\pi ]$, since the case $\theta \in (\pi ,2\pi )$ can be proved similarly. Using the identity

$$\begin{aligned} \frac{(1-\xi )^2}{\xi } = \frac{1}{\xi }+\xi -2= e^{-\mathrm {i}\theta }+e^{\mathrm {i}\theta }-2 = 2\cos \theta -2, \end{aligned}$$

we have

$$\begin{aligned} \arg ( {(1-\xi )^2}/{\xi }) = \arg (2\cos \theta -2) = -\pi . \end{aligned}$$

Moreover, we have the expansion [49, equation (13.1)]

4.5 $$\begin{aligned}&\frac{\mathrm {Li}_{\alpha -1}(\xi )}{\varGamma (2-\alpha )} \nonumber \\&\quad =(-2\pi \mathrm {i})^{\alpha -2}\sum _{k=0}^\infty \left( k+1-\tfrac{\theta }{2\pi }\right) ^{\alpha -2} + (2\pi \mathrm {i})^{\alpha -2}\sum _{k=0}^\infty \left( k+\tfrac{\theta }{2\pi }\right) ^{\alpha -2} \nonumber \\&\quad =(2\pi )^{\alpha -2} \left( \cos ((2-\alpha )\tfrac{\pi }{2})(A(\theta )+B(\theta )) - {\mathrm i}\sin ((2-\alpha )\tfrac{\pi }{2}) (A(\theta )-B(\theta )) \right) ,\nonumber \\ \end{aligned}$$

where

$$\begin{aligned} A(\theta )=\sum _{k=0}^\infty \left( k+\tfrac{\theta }{2\pi }\right) ^{\alpha -2} \quad \text {and}\quad B(\theta )=\sum _{k=0}^\infty \left( k+1-\tfrac{\theta }{2\pi }\right) ^{\alpha -2}. \end{aligned}$$

Both series converge for $\alpha \in (0,1)$. Since for $\theta \in (0,\pi ]$, $(k+\tfrac{\theta }{2\pi } )^{\alpha -2}> (k+1-\tfrac{\theta }{2\pi } )^{\alpha -2}>0$, there holds

$$\begin{aligned} \frac{A(\theta )-B(\theta )}{A(\theta )+B(\theta )} \in (0,1), \end{aligned}$$

and we deduce

$$\begin{aligned} \arg ( \mathrm {Li}_{\alpha -1}(\xi ) ) \in [-\pi ,-\pi +\alpha \pi /2)\quad \text {for}~~ \xi =e^{-\mathrm {i}\theta },~\theta \in (0,\pi ]. \end{aligned}$$

Therefore, we have

$$\begin{aligned} \arg \Big (\frac{(1-\xi )^2}{\xi }\mathrm {Li}_{\alpha -1}(\xi ) \Big )=\arg ( \mathrm {Li}_{\alpha -1}(\xi ) ) +\arg ( {(1-\xi )^2}/{\xi })\in [0,\alpha \pi /2). \end{aligned}$$

This completes the proof of the lemma. $\square$

### Lemma 5

For the function $\delta (\xi )$ defined by (4.4), there holds

$$\begin{aligned} (1-\xi )(1+\xi )\delta ^\prime (\xi ) = d(\xi ) \delta (\xi ) \end{aligned}$$

with

$$\begin{aligned} d(\xi )= \left\{ \begin{array}{ll}\displaystyle (1+\xi )\left( -2+\frac{1-\xi }{\xi }\frac{\mathrm {Li}_{\alpha -2}(\xi )-\mathrm {Li}_{\alpha -1}(\xi )}{\mathrm {Li}_{\alpha -1}(\xi )}\right) , &{}\quad \alpha \in (0,1),\\ \displaystyle (1+\xi )\left( -3+\frac{1-\xi }{\xi }\frac{\mathrm {Li}_{\alpha -3}(\xi )-\mathrm {Li}_{\alpha -2}(\xi )}{\mathrm {Li}_{\alpha -2}(\xi )}\right) + (\xi -1), &{}\quad \alpha \in (1,2). \end{array}\right. \end{aligned}$$

where $d(\xi )$ is uniformly bounded on ${\mathbb D}'$.

### Proof

It suffices to consider the case $\alpha \in (0,1)$, while the other case follows analogously. Since $\mathrm {Li}_{\alpha -1}(\xi )$ is analytic, by termwise differentiation, $\mathrm {Li}^\prime _{\alpha -1}(\xi )=\xi ^{-1}\mathrm {Li}_{\alpha -2}(\xi )$. Thus, with $c_\alpha =1/\varGamma (2-\alpha )$, we have

$$\begin{aligned} \delta ^\prime (\xi ) = c_\alpha \Big ({-}\frac{2(1-\xi )}{\xi }\mathrm {Li}_{\alpha -1}(\xi ) -\frac{(1-\xi )^2}{\xi ^2}\mathrm {Li}_{\alpha -1}(\xi ) +\frac{(1-\xi )^2}{\xi ^2}\mathrm {Li}_{\alpha -2}(\xi )\Big ), \end{aligned}$$

from which the expression of $d(\xi )$ follows. By using the asymptotic expansion (see [49, equation (9.3)] or [14, Theorem 1])

4.6 $$\begin{aligned} \mathrm {Li}_{p}(e^{-\mathrm {i}\theta })=\varGamma (1-p)(\mathrm {i}\theta )^{p-1} + o(\theta ^{p}), \quad \hbox {as } \theta \rightarrow 0, \end{aligned}$$

we deduce

$$\begin{aligned} \begin{array}{c} \lim \limits _{\xi \rightarrow 1}\\ {\xi \in {\mathbb D}'} \end{array}\frac{1-\xi }{\xi }\frac{\mathrm {Li}_{\alpha -2}(\xi )-\mathrm {Li}_{\alpha -1}(\xi )}{\mathrm {Li}_{\alpha -1}(\xi )} =\frac{\varGamma (3-\alpha )}{\varGamma (2-\alpha )} = 2-\alpha . \end{aligned}$$

Hence, $d(\xi )$ is bounded if $\xi =e^{-{\mathrm i}\theta }$ is close to 1. Meanwhile, if $\xi =e^{-{\mathrm i}\theta }$ and $\theta$ is away from the two end-points of the interval $(0,2\pi )$, then (4.5) implies that $|\mathrm{Li}_{ \alpha -1}(\xi )|$ has a positive lower bound and $|\mathrm{Li}_{\alpha -2}(\xi )|$ has an upper bound. Hence $d(\xi )$ is bounded.

$$\square $$

Now we can give the discrete maximal regularity for the L1 scheme (4.1).

### Theorem 7

Let *X* be a UMD space, $0<\alpha <1$ or $1<\alpha <2$, and let *A* be an *R*-sectorial operator on *X* of angle $\alpha \pi /2$. Then the L1 scheme (4.3) satisfies the following discrete maximal regularity

$$\begin{aligned}&\Vert (\bar{\partial }_\tau ^\alpha u^n)_{n=1}^N\Vert _{\ell ^p(X)} + \Vert (Au^n)_{n=1}^N\Vert _{\ell ^p(X)} \le \left\{ \begin{aligned} c_{p,X}c_R\Vert (f^n)_{n=1}^N\Vert _{\ell ^p(X)},\;\hbox {if}\; 0<\alpha<1,\\ c_{p,X}c_R\Vert (f^n)_{n=0}^N\Vert _{\ell ^p(X)},\;\hbox {if}\; 1<\alpha <2, \end{aligned} \right. \end{aligned}$$

where the constant $c_{p,X}$ is independent of *N*, $\tau$ and *A*, and $c_R$ denotes the *R*-bound of the set of operators $\{zR(z;A) :z\in \varSigma _{\alpha \pi /2}\}$.

### Proof

First we consider the case $0<\alpha <1$. Upon setting $f^0=0$, Lemmas 3 and 4 yield

$$\begin{aligned} (\bar{\partial }_\tau ^\alpha u)(\xi ) = M(\xi )f(\xi )\quad \hbox {with } M(\xi )= \tau ^{-\alpha }\delta (\xi )\left( \tau ^{-\alpha }\delta (\xi )-A\right) ^{-1}, \end{aligned}$$

where $\delta (\xi )$ is defined by (4.4). By Lemma 4, we have

$$\begin{aligned} \{M(\xi ):\xi \in \mathbb {D}^\prime \}\subset \{z R(z;A):z\in \varSigma _{\alpha \pi /2}\}, \end{aligned}$$

where the latter set is *R*-bounded by assumption. Meanwhile,

$$\begin{aligned} (1-\xi )(1+\xi )M^\prime (\xi ) = d(\xi )M(\xi )-d(\xi )M(\xi )^2, \end{aligned}$$

where, by Lemma 5, $d(\xi )$ is uniformly bounded on $\mathbb {D}^\prime$. By Lemma 1, the set $\{(1-\xi )(1+\xi )M^\prime (\xi ):\xi \in \mathbb {D}^\prime \}$ is *R*-bounded. Thus we deduce from Theorem 4 the desired assertion.

Next we consider the case of $1<\alpha <2$. In this case, we let $g^0=0$ and $g^n=f^n$, $n\ge 1$, to obtain

$$\begin{aligned} (\bar{\partial }_\tau ^\alpha u)(\xi )= \tfrac{1}{2} \xi M(\xi )f(\xi ) + \tfrac{1}{2} M(\xi ) g(\xi ), \end{aligned}$$

with $M(\xi )= \tau ^{-\alpha }\delta (\xi )(\tau ^{-\alpha }\delta (\xi )-A)^{-1}$. In view of the relation $\delta (\xi )=\frac{2}{\varGamma (3-\alpha )} \frac{1-\xi }{1+\xi }\psi (\xi )$, by Lemma 4 and since the function $(1-\xi )/(1+\xi )$ maps $\mathbb {D}^\prime$ into the imaginary axis, we deduce

$$\begin{aligned} \{M(\xi ):\xi \in \mathbb {D}^\prime \}\subset \{\lambda R(\lambda ;A):\lambda \in \varSigma _{\alpha \pi /2}\}. \end{aligned}$$

The rest of the proof follows like before, using Lemma 5. $\square$

### Remark 3

For the model (1.3) with $\alpha \in (0,1)$, the piecewise constant discontinuous Galerkin (PCDG) method in [39] leads to a time-stepping scheme identical with the L1 scheme. The PCDG is given by: find $u^n$ such that

$$\begin{aligned} \int _{t_{n-1}}^{t_n}\partial _t^{\alpha }u(s) ds = \int _{t_{n-1}}^{t_n}Au^n(s)ds + \int _{t_{n-1}}^{t_n}f(s)ds,\quad n=1,2,\ldots ,N. \end{aligned}$$

By letting $f^n=\tau ^{-1}\int _{t_{n-1}}^{t_n}f(s)ds$, we obtain

$$\begin{aligned} \tau ^{-1}\int _{t_{n-1}}^{t_n}\partial _t^{\alpha }u(s)ds = Au^n + f^n,\quad n=1,\ldots ,N. \end{aligned}$$

Next we derive the explicit expression for the discrete approximation $\bar{\partial }_\tau ^\alpha u^n$

$$\begin{aligned} \bar{\partial }_\tau ^\alpha u^n = \tau ^{-1}\int _{t_{n-1}}^{t_n}\partial _t^\alpha u(s)ds =\tau ^{-\alpha }\sum _{j=1}^n\beta _{n-j}u^j, \end{aligned}$$

where $\beta _0=1$ and $\beta _j=(j+1)^{1-\alpha }-2j^{1-\alpha }+(j-1)^{1-\alpha }$, $j=1,2,\ldots$. With the weights $b_j$ in (4.2), we have $\beta _j=b_j-b_{j-1}$, for $j=1,2,\ldots$, and $\beta _0=b_0$. Hence, the PCDG approximation $\bar{\partial }_\tau ^\alpha u^n$ reads

$$\begin{aligned} \bar{\partial }_\tau ^\alpha u^n = \tau ^{-\alpha }b_0u^n + \tau ^{-\alpha }\sum _{j=1}^{n-1}(b_j-b_{j-1})u^{n-j}. \end{aligned}$$

Thus it is identical with the L1 scheme, and Theorem 7 applies.

## Explicit Euler method

Now we analyze the explicit Euler method for discretizing (1.3) in time:

5.1 $$\begin{aligned} \bar{\partial }_\tau ^\alpha u^n = Au^{n-1}+f^{n-1}, \quad n\ge 1, \end{aligned}$$

where the approximation $\bar{\partial }_\tau ^\alpha u^n$ denotes the BE approximation (3.2). A variant of the scheme was analyzed in [50]. By multiplying (5.1) by $\xi ^n$ and summing up the results for $n=1,2,\dots ,$ we obtain

$$\begin{aligned} (\tau ^{-\alpha }\delta (\xi )-A)u(\xi ) = f(\xi )\quad \hbox {and}\quad (\bar{\partial }_\tau ^\alpha u)(\xi ) = \tau ^{-\alpha }\xi \delta (\xi )u(\xi ), \end{aligned}$$

with

$$\begin{aligned} \delta (\xi ) =\tfrac{(1-\xi )^\alpha }{\xi }. \end{aligned}$$

Recall that the numerical range *S*(*A*) of an operator *A* is defined by [42, pp. 12]

$$\begin{aligned} S(A) = \{\langle x^*,Ax\rangle : x\in X,x^*\in X^*, \Vert x\Vert _X=\Vert x^*\Vert _{X^*}=\langle x^*,x\rangle =1\}. \end{aligned}$$

We denote by $r(A)=\sup _{z\in S(A)}|z|$ the radius of the numerical range *S*(*A*), known as numerical radius. Recall that [42, Theorem 3.9, Chapter 1, pp. 12]

5.2 $$\begin{aligned} \Vert R(z;A)\Vert _{\mathcal {B}(X)}\le \mathrm {dist}(z,\overline{S(A)})^{-1},\quad \forall \, z\in \mathbb {C}{\setminus } \overline{S(A)}, \end{aligned}$$

where $\overline{S(A)}$ denotes of the closure of *S*(*A*) in $\mathbb {C}$, and $\mathrm {dist} (z,\overline{S(A)})$ is the distance of *z* from $\overline{S(A)}$.

The next theorem gives the maximal $\ell ^p$-regularity of the explicit Euler method (5.1), if $\tau ^\alpha r(A)$ is smaller than some given positive constant.

### Theorem 8

Let *X* be a UMD space, $0<\alpha <1$ or $1<\alpha <2$, and let *A* be an *R*-sectorial operator of angle $\alpha \pi /2$ such that $S(A)\subset {\mathbb C}\backslash \varSigma _{\varphi }$ for some $\varphi \in (\alpha \pi /2,\pi ]$. Then, under the condition (for small $\epsilon >0$)

5.3 $$\begin{aligned} \tau ^\alpha r(A)\le 2^\alpha \bigg [\sin \bigg (\frac{\varphi -\alpha \pi /2}{2-\alpha }\bigg )\bigg ]^\alpha -\epsilon , \end{aligned}$$

the scheme (5.1) satisfies the following discrete maximal regularity

$$\begin{aligned} \Vert (\bar{\partial }_\tau ^\alpha u^n)_{n=1}^N\Vert _{\ell ^p(X)} +\Vert (Au^n)_{n=1}^{N-1}\Vert _{\ell ^p(X)}\le c_{p,X}(1+c_R)\Vert (f^n)_{n=0}^{N-1}\Vert _{\ell ^p(X)}, \end{aligned}$$

where the constant $c_{p,X}$ depends only on $\epsilon$, $\varphi$ and $\alpha$ (independent of $\tau$ and *A*), and $c_R$ denotes the *R*-bound of the set $\{zR(z;A) :z\in \varSigma _{\alpha \pi /2}\}$.

### Proof

For $\xi =e^{\mathrm {i}\theta }$, $\theta \in (0,2\pi )$, we have

$$\begin{aligned} \frac{\delta (e^{\mathrm {i}\theta })}{\tau ^\alpha }=\frac{2^\alpha [\sin (\theta /2)]^\alpha }{\tau ^\alpha } e^{\mathrm {i}[-\alpha \pi /2-(1-\alpha /2)\theta ]}, \end{aligned}$$

which is a parametric curve contained in the sector ${\mathbb C}\backslash \overline{\varSigma }_{\alpha \pi /2}$. Let $\varGamma =\{\tau ^{-\alpha }\delta (e^{\mathrm {i}\theta }):\theta \in (0,2\pi )\}$. It suffices to prove that the family of operators $\{zR(z;A):z\in \varGamma \}$ is *R*-bounded. Since $\{zR(z;A):z\in \varSigma _{\alpha \pi /2}\}$ is *R*-bounded, by Lemma 2, we have $\{zR(z;A):z\in \varGamma \cap \varSigma _{\phi }\}$ is *R*-bounded for some $\phi \in (\alpha \pi /2,\varphi ]$, where $\phi$ depends on $c_R$ and $\alpha$. It remains to prove that $\{zR(z;A):z\in \varGamma \backslash \varSigma _{\phi }\}$ is also *R*-bounded. Note that $\mathrm{arg}(\tau ^{-\alpha }\delta (e^{\mathrm {i}\theta })) \in {\mathbb C}\backslash \varSigma _{\varphi }$ is equivalent to

5.4 $$\begin{aligned} \frac{\varphi -\alpha \pi /2}{1-\alpha /2}<\theta < 2\pi -\frac{\varphi -\alpha \pi /2}{1-\alpha /2}. \end{aligned}$$

Meanwhile, since for $\theta \in (0,\pi )$, $|\delta (e^{\mathrm {i}\theta })|=2^\alpha [\sin (\theta /2)]^\alpha$ is strictly monotonically increasing in $\theta$, for such $\theta$ satisfying (5.4), there holds

$$\begin{aligned} \bigg |\frac{\delta (e^{\mathrm {i}\theta })}{\tau ^\alpha }\bigg | \ge \frac{2^\alpha \big [\sin \big (\frac{\varphi -\alpha \pi /2}{2-\alpha }\big )\big ]^\alpha }{\tau ^\alpha }. \end{aligned}$$

If (5.3) is satisfied, then

5.5 $$\begin{aligned} (1-\epsilon _{\alpha ,\varphi })|\delta (e^{\mathrm {i}\theta }) |\ge \tau ^\alpha r(A) \end{aligned}$$

for some $\epsilon _{\alpha ,\varphi }>0$. Now consider the curve $\varGamma _0:=\{\delta (e^{\mathrm {i}\theta }): \theta \in (0,2\pi )\}$ and the closed region $D_0:=\{s\varGamma _0:s\in [0,1]\}$, which are fixed (and independent of $\tau$). Since $\overline{S(A)}\subset {\mathbb C}\backslash \varSigma _\varphi$, it follows from (5.5) that

$$\begin{aligned} \mathrm{dist}(z,\tau ^{\alpha }\overline{S(A)})\ge \mathrm{dist}(\varGamma _0\backslash \varSigma _{\phi }, (1-\epsilon _{\alpha ,\varphi })D_0\backslash \varSigma _\varphi ) \ge C^{-1} \quad \hbox { for }z\in \varGamma _0\backslash \varSigma _{\phi }. \end{aligned}$$

where the constant *C* depends on the parameters $\epsilon$, $\alpha$, $\varphi$ and $\phi$, but is independent of $\tau$ (since both $\varGamma _0\backslash \varSigma _{\phi }$ and $(1-\epsilon _{\alpha ,\varphi })D_0\backslash \varSigma _\varphi$ are fixed closed subsets of ${\mathbb C}$, independent of $\tau$). Since $\varGamma =\tau ^{-\alpha }\varGamma _0$, the last inequality yields (via scaling)

$$\begin{aligned} \mathrm{dist}(z,\overline{S(A)})\ge \tau ^{-\alpha } C^{-1} \quad \hbox { for }z\in \varGamma \backslash \varSigma _{\phi }. \end{aligned}$$

Hence there exists a finite number of balls $B(z_j,\rho )$ of radius $\rho =\frac{1}{4}\tau ^{-\alpha } C^{-1}$, $z_j\in \varGamma$, which can cover $\varGamma \backslash \varSigma _{\phi }$, and further, the number of balls is bounded by a constant which depends only the parameters $\epsilon$, $\alpha$, $\varphi$ and $\phi$, independent of $\tau$ and *A*. For each ball $B(z_j,\rho )$, $\{zR(z;A):z\in B(z_j,\rho )\}$ is *R*-bounded and its *R*-bound is at most (see Lemma 6 below)

$$\begin{aligned} \sup _{z\in B(z_j,\rho )} 2|z|\Vert R(z;A)\Vert _{\mathcal {B}(X)} \le \sup _{z\in B(z_j,\rho )} 2|z|\, \mathrm{dist}(z,\overline{S(A)})^{-1} \le C, \end{aligned}$$

where we have used the estimate (5.2) in the first inequality. Then Lemma 1 (iii) implies that $\{zR(z;A):z\in \varGamma \backslash \varSigma _{\phi }\}$ is also *R*-bounded. $\square$

### Remark 4

The constant in condition (5.3) is sharp. The scaling factor $\tau ^\alpha$ is one notable feature of the model (1.3), and for $\alpha \in (0,1)$, the exponent $\alpha$ agrees with that in the stability condition in [50]. Hence, the smaller the fractional order $\alpha$ is, the smaller the step size $\tau$ should be taken.

### Remark 5

The condition (5.3) covers bounded operators, e.g., finite element approximations of a self-adjoint second-order elliptic operator. For a self-adjoint discrete approximation, the numerical range *S*(*A*) is the closed interval spanned by the largest and smallest eigenvalues, but in general, the numerical range *S*(*A*) has to be approximated [19, Section 5.6].

### Lemma 6

(*R*-boundedness of operator-valued analytic functions) If the function $F:\overline{B}(z_0,\rho )\rightarrow \mathcal {B}(X)$ is analytic in a neighborhood of the ball $\overline{B}(z_0,\rho )$, centered at $z_0$ with radius $\rho$, then the set of operators $\{F(z):\lambda \in B(z_0,\rho /2)\}$ is *R*-bounded on *X*, and its *R*-bound is at most

$$\begin{aligned} 2\sup _{z\in B(z_0,\rho )}\Vert F(z)\Vert _{\mathcal {B}(X)}. \end{aligned}$$

### Proof

The analyticity implies the existence of a power series expansion

$$\begin{aligned} F(z)=\sum _{n=0}^\infty \frac{F_n}{n!}(z-z_0)^n \end{aligned}$$

where $F_n$, $n=0,1,2,\dots$ are bounded linear operators on *X* and the series converges absolutely in $B(z_0,\rho )$. Moreover, by Cauchy’s integral formula,

$$\begin{aligned} \Vert F_n\Vert _{\mathcal {B}(X)}= \bigg \Vert \frac{1}{2\pi \mathrm {i}}\int _{\partial B(z_0,\rho )}\frac{n! F(z)}{(z-z_0)^{n+1}} dz\bigg \Vert _{\mathcal {B}(X)} \le \rho ^{-n}n!\sup _{z\in B(z_0,\rho )}\Vert F(z)\Vert _{\mathcal {B}(X)}. \end{aligned}$$

Hence, for $z_j\in B(z_0,\rho /2)$ and $u_j\in X$, $j=1,2,\dots ,m$, Minkowski’s inequality implies

$$\begin{aligned} \int _0^1\bigg \Vert \sum _{j=1}^m r_j(s)F(z_j)u_j\bigg \Vert _X ds&\le \sum _{n=0}^\infty \frac{(\rho /2)^n}{n!} \int _0^1\bigg \Vert \sum _{j=1}^mr_j(s)\bigg (\frac{z_j-z_0}{\rho /2}\bigg )^n F_nu_j\bigg \Vert _X ds \\&\le 2\sum _{n=0}^\infty \frac{(\rho /2)^n}{n!} \int _0^1\bigg \Vert \sum _{j=1}^mr_j(s)F_nu_j\bigg \Vert _X ds \\&\le 2\sum _{n=0}^\infty \frac{(\rho /2)^n\Vert F_n\Vert _{\mathcal {B}(X)} }{n!} \int _0^1\bigg \Vert \sum _{j=1}^mr_j(s)u_j\bigg \Vert _X ds \\&\le 2\sum _{n=0}^\infty 2^{-n}\sup _{z\in B(z_0,\rho )}\Vert F(z)\Vert _{\mathcal {B}(X)} \int _0^1\bigg \Vert \sum _{j=1}^mr_j(s)u_j\bigg \Vert _X ds \\&\le 4 \sup _{z\in B(z_0,\rho )}\Vert F(z)\Vert _{\mathcal {B}(X)} \int _0^1\bigg \Vert \sum _{j=1}^mr_j(s)u_j\bigg \Vert _X ds, \end{aligned}$$

where the second line follows from [30, Proposition 2.5]. This shows that the family of operators $\{F(z):z\in B(z_0,\rho /2)\}$ is *R*-bounded. $\square$

## Fractional Crank–Nicolson method

By the fractional Crank–Nicolson method, we mean the following scheme:

6.1 $$\begin{aligned} \bar{\partial }_\tau ^\alpha u^n = (1-\tfrac{\alpha }{2})Au^{n}+\tfrac{\alpha }{2}Au^{n-1}+(1-\tfrac{\alpha }{2})f^{n}+\tfrac{\alpha }{2}f^{n-1}, \end{aligned}$$

where the approximation $\bar{\partial }_\tau ^\alpha u^n$ denotes the BE approximation (3.2). When $\alpha =1$, (6.1) coincides with the standard Crank–Nicolson method. For any $0<\alpha <2$, one can verify that it is second-order in time, provided that the solution is sufficiently smooth [22]. By multiplying (5.1) by $\xi ^n$ and summing up the results for $n=1,2,\dots ,$ we obtain

$$\begin{aligned}&(\tau ^{-\alpha }\delta (\xi )-A)u(\xi ) = f(\xi ) \\&(\bar{\partial }_\tau ^\alpha u)(\xi ) = \left( 1-\tfrac{\alpha }{2}+\tfrac{\alpha }{2}\xi \right) \tau ^{-\alpha }\delta (\xi )u(\xi ), \end{aligned}$$

with

$$\begin{aligned} \delta (\xi ) =\frac{(1-\xi )^\alpha }{1-\frac{\alpha }{2} +\frac{\alpha }{2}\xi }. \end{aligned}$$

First, we prove the maximal $\ell ^p$-regularity for (6.1) in the case $0<\alpha <1$.

### Theorem 9

Let *X* be a UMD space, $0<\alpha <1$, and let *A* be an *R*-sectorial operator on *X* of angle $\alpha \pi /2$. Then the scheme (6.1) satisfies the following discrete maximal regularity

$$\begin{aligned} \Vert (\bar{\partial }_\tau ^\alpha u^n)_{n=1}^N\Vert _{\ell ^p(X)} +\Vert (Au^n)_{n=1}^N\Vert _{\ell ^p(X)}\le c_{p,X}c_R\Vert (f^n)_{n=0}^{N}\Vert _{\ell ^p(X)}, \end{aligned}$$

where the constant $c_{p,X}$ depends only on $\alpha$ (independent of $\tau$ and *A*), and $c_R$ denotes the *R*-bound of the set of operators $\{zR(z;A) :z\in \varSigma _{\alpha \pi /2}\}$.

### Proof

It suffices to prove that the family of operators $\big \{\tau ^{-\alpha }\delta (\xi )(\tau ^{-\alpha }\delta (\xi )-A)^{-1}:\xi \in {\mathbb D}'\big \}$ is *R*-bounded. In fact, for $\xi =e^{\mathrm {i}\theta }$, $\theta \in (0,2\pi )$, we have

$$\begin{aligned} \frac{\delta (e^{\mathrm {i}\theta })}{\tau ^\alpha }=\frac{2^\alpha [\sin (\theta /2)]^\alpha }{\tau ^\alpha \rho (\theta )} e^{\mathrm {i}(-\frac{\alpha }{2}\pi +\frac{\alpha }{2}\theta -\psi (\theta ))}, \end{aligned}$$

where the functions $\rho (\theta )$ and $\psi (\theta )$ are defined respectively by

6.2 $$\begin{aligned}&\rho (\theta ) :=\sqrt{(1-\tfrac{\alpha }{2})^2+\tfrac{\alpha ^2}{4}+\alpha (1-\tfrac{\alpha }{2})\cos \theta }, \end{aligned}$$

and

6.3 $$\begin{aligned}&\psi (\theta ) :=\arg \Big (1-\frac{\alpha }{2}+\frac{\alpha }{2}\cos \theta +\mathrm {i} \frac{\alpha }{2}\sin \theta \Big ) =\arctan \frac{\frac{\alpha }{2}\sin \theta }{1-\frac{\alpha }{2}+\frac{\alpha }{2}\cos \theta }. \end{aligned}$$

It is straightforward to compute

$$\begin{aligned} \frac{\alpha }{2}-\psi '(\theta ) =\frac{\frac{\alpha }{2}(1-\alpha )(1-\frac{\alpha }{2})(1-\cos \theta )}{(1-\frac{\alpha }{2}+\frac{\alpha }{2}\cos \theta )^2+\frac{\alpha ^2}{4}\sin ^2\theta } \ge 0. \end{aligned}$$

Thus $\frac{\alpha }{2}\theta -\psi (\theta )$ is an increasing function of $\theta$, taking values from 0 to $\alpha \pi$ as $\theta$ changes from 0 to $2\pi$. Thus $\tau ^{-\alpha }\delta (e^{\mathrm {i}\theta })\in \varSigma _{\alpha \pi /2}$, and by Lemma 1, the set $\{(1-\frac{\alpha }{2}+\frac{\alpha }{2} \xi )\tau ^{-\alpha }\delta (\xi )(\tau ^{-\alpha }\delta (\xi )-A)^{-1}:\xi \in {\mathbb D}'\}$ is *R*-bounded. $\square$

Let the function $\psi$ be defined in (6.3), and $\theta _\varphi \in (0,\pi )$ be the unique root of the equation

6.4 $$\begin{aligned} \psi (\theta _\varphi )-\frac{\alpha }{2}\theta _\varphi =\varphi -\frac{\alpha \pi }{2}. \end{aligned}$$

Then we have the following result for the case $1<\alpha <2$.

### Theorem 10

Let *X* be a UMD space, $1<\alpha <2$, and let *A* be an *R*-sectorial operator on *X* of angle $\alpha \pi /2$ such that $S(A)\subset {\mathbb C}\backslash \varSigma _{\varphi }$ for some $\varphi \in (\alpha \pi /2,\pi )$. Then, under the condition (for small $\epsilon >0$)

6.5 $$\begin{aligned} \tau ^\alpha r(A)\le \frac{2^\alpha [\sin (\theta _\varphi /2)]^\alpha }{ \rho (\theta _\varphi )} -\epsilon , \end{aligned}$$

the scheme (6.1) satisfies the following discrete maximal regularity

$$\begin{aligned} \Vert (\bar{\partial }_\tau ^\alpha u^n)_{n=1}^N\Vert _{\ell ^p(X)} +\Vert (Au^n)_{n=1}^N\Vert _{\ell ^p(X)}\le c_{p,X}(1+c_R)\Vert (f^n)_{n=0}^{N}\Vert _{\ell ^p(X)}, \end{aligned}$$

where the constant $c_{p,X}$ depends only on $\epsilon$, $\varphi$ and $\alpha$ (independent of $\tau$ and *A*), and $c_R$ denotes the *R*-bound of the set $\{zR(z;A) :z\in \varSigma _{\alpha \pi /2}\}$.

### Proof

If $$1<\alpha <2$$, then

$$\begin{aligned} \frac{\alpha }{2}-\psi '(\theta ) =\frac{\frac{\alpha }{2}(1-\alpha )(1-\frac{\alpha }{2})(1-\cos \theta )}{(1-\frac{\alpha }{2}+\frac{\alpha }{2}\cos \theta )^2+\frac{\alpha ^2}{4}\sin ^2\theta } \le 0. \end{aligned}$$

Hence, $\frac{\alpha }{2}\theta -\psi (\theta )$ is a decreasing function of $\theta$, taking values from 0 to $\alpha \pi -2\pi$ as $\theta$ changes from 0 to $2\pi$. Thus $\tau ^{-\alpha }\delta (e^{\mathrm {i}\theta })\in {\mathbb C}\backslash \varSigma _{\alpha \pi /2}$. With $\varGamma =\{\tau ^{-\alpha } \delta (e^{\mathrm {i}\theta }): \theta \in (0,2\pi )\}$, it suffices to show that $\{zR(z;A):z\in \varGamma \}$ is *R*-bounded. Since $\{zR(z;A):z\in \varSigma _{\alpha \pi /2}\}$ is *R*-bounded, by Lemma 2, $\{zR(z;A):z\in \varGamma \cap \varSigma _{\phi }\}$ is *R*-bounded for some $\phi \in (\alpha \pi /2,\pi )$, where $\phi$ depends on $c_R$ and $\alpha$. It remains to prove that $\{zR(z;A):z\in \varGamma \backslash \varSigma _{\phi }\}$ is also *R*-bounded. However, $\mathrm{arg}(\tau ^{-\alpha }\delta (e^{\mathrm {i}\theta }))\in {\mathbb C}\backslash \varSigma _{\varphi }$ is equivalent to

6.6 $$\begin{aligned} \theta _\varphi<\theta < 2\pi -\theta _\varphi , \end{aligned}$$

where $\theta _\varphi$ is the unique root of equation (6.4). Meanwhile, for $\theta \in (0,\pi )$, $|\delta (e^{\mathrm {i}\theta })|=2^\alpha [\sin (\theta /2)]^\alpha /\rho (\theta )=2^\alpha \sin (\theta /2)^{\alpha -1}\cdot \sin (\theta /2)/\rho (\theta )$ is monotonically increasing. Hence, for any $\theta$ satisfying (6.6), we have

$$\begin{aligned} \bigg |\frac{\delta (e^{\mathrm {i}\theta })}{\tau ^\alpha }\bigg | \ge \frac{2^\alpha [\sin (\theta _\varphi /2)]^\alpha }{ \rho (\theta _\varphi )\tau ^\alpha }. \end{aligned}$$

If (6.5) is satisfied then for some positive constant $\epsilon _{\alpha ,\varphi }$,

$$\begin{aligned} (1-\epsilon _{\alpha ,\varphi })\bigg |\frac{\delta (e^{\mathrm {i}\theta })}{\tau ^\alpha }\bigg |\ge r(A). \end{aligned}$$

By repeating the argument in Theorem 8, we deduce $\mathrm{dist}(z,\overline{S(A)})\ge \tau ^{-\alpha } C^{-1}$ for $z\in \varGamma \backslash \varSigma _{\phi }$, where *C* is some constant which may depend on $\epsilon$, $\alpha$, $\varphi$ and $\phi$, but is independent of $\tau$. Hence, there exists a finite number of balls $B(z_j,\rho )$ of radius $\rho =\frac{1}{4}\tau ^{-\alpha } C^{-1}$, $z_j\in \varGamma$, which can cover $\varGamma \backslash \varSigma _{\phi }$, and the number of balls is bounded by a constant which depends only on $\epsilon$, $\alpha$, $\varphi$ and $\phi$, independent of $\tau$ and *A*. By Lemma 6, for each ball $B(z_j,\rho )$, $\{zR(z;A):z\in B(z_j,\rho )\}$ is *R*-bounded and its *R*-bound is at most

$$\begin{aligned} 2\sup _{z\in B(z_j,\rho )}|z|\Vert R(z;A)\Vert _{\mathcal {B}(X)} \le 2\sup _{z\in B(z_j,\rho )}|z|\, \mathrm{dist}(z,\overline{S(A)})^{-1} \le C. \end{aligned}$$

Then Lemma 1 (iii) implies that $\{zR(z;A):z\in \varGamma \backslash \varSigma _{\phi }\}$ is also *R*-bounded. $\square$

## Inhomogeneous initial condition

In this section, we consider maximal $\ell ^p$-regularity for the problem

7.1 $$\begin{aligned} \partial _t^\alpha u(t) = A u(t), \quad t>0 \end{aligned}$$

with nontrivial initial conditions:

7.2 $$\begin{aligned} \begin{aligned}&u(0)=v,\qquad&\quad \hbox {(for }0<\alpha<1),\\&u(0)=v,\,\,\, \partial _tu(0)= w,&\quad \hbox {(for }1<\alpha <2). \end{aligned} \end{aligned}$$

We focus on the BE scheme since other schemes can be analyzed similarly. For (7.2), the BE scheme reads [21, 22]: with $$u^0=v $$, find $$u^n$$ such that

7.3 $$\begin{aligned} \begin{aligned} \bar{\partial }_\tau ^\alpha (u-v)^n&= A u^n,\quad n=1,2,\dots \quad \hbox {(for }0<\alpha<1),\\ \bar{\partial }_\tau ^\alpha (u-v-t w)^n&= A u^n,\quad n=1,2,\dots \quad \hbox {(for }1<\alpha <2), \end{aligned} \end{aligned}$$

where $\bar{\partial }_\tau ^\alpha$ denotes the BE convolution quadrature (3.2).

We shall need the scaled $L^p$-norm and weak $L^p$-norm (cf. [9, section 1.3])

7.4 $$\begin{aligned}&\Vert (u^n)_{n=1}^N\Vert _{L^p(X)} :=\bigg (\tau \sum _{n=1}^N\Vert u^n\Vert _X^p\bigg )^{\frac{1}{p}}, \end{aligned}$$

7.5 $$\begin{aligned}&\Vert (u^n)_{n=1}^N\Vert _{L^{p,\infty }(X)} :=\sup _{\lambda>0} \lambda |\{n\ge 1: \Vert u^n\Vert _X>\lambda \}|^{\frac{1}{p}}\tau ^{\frac{1}{p}}. \end{aligned}$$

The main result of this section is the following theorem.

### Theorem 11

Let *X* be a Banach space, $0<\alpha <1$, and let *A* be a sectorial operator on *X* of angle $\alpha \pi /2$. Then the BE scheme (7.3) has the following maximal $\ell ^p$-regularity

$$\begin{aligned}&\Vert (\bar{\partial }_\tau ^\alpha u^n)_{n=1}^N\Vert _{L^p(X)}+ \Vert (Au^n)_{n=1}^N\Vert _{L^p(X)} \le c_{p}\Vert v\Vert _{(X,D(A))_{1-\frac{1}{p\alpha },p}},&p\in (1/\alpha ,\infty ], \\&\Vert (\bar{\partial }_\tau ^\alpha u^n)_{n=1}^N\Vert _{L^{p,\infty }(X)}+ \Vert (Au^n)_{n=1}^N\Vert _{L^{p,\infty }(X)} \le c_{p}\Vert v\Vert _{X},&p=1/\alpha , \\&\Vert (\bar{\partial }_\tau ^\alpha u^n)_{n=1}^N\Vert _{L^{p}(X)} + \Vert (Au^n)_{n=1}^N\Vert _{L^{p}(X)} \le c_{p}\Vert v\Vert _{X},&p\in [1,1/\alpha ), \end{aligned}$$

where the constant $c_{p}$ depends on the bound of the set of operators $\{zR(z;A) :z\in \varSigma _{\alpha \pi /2}\}$, independent of *N*, $\tau$ and *A*.

### Proof

By multiplying both sides of (7.3) by $\xi ^n$ and summing over *n*, we have

$$\begin{aligned} \sum _{n=1}^\infty \xi ^n \bar{\partial }_\tau ^\alpha (u-v)^n - \sum _{n=1}^\infty Au^n\xi ^n = 0. \end{aligned}$$

Let $u(\xi )=\sum _{n=1}^\infty u^n\xi ^n$. Then by repeating the argument in the proof of Theorem 5, we have (with $\delta (\xi )=1-\xi$)

$$\begin{aligned} Au(\xi ) = A(\tau ^{-\alpha }\delta (\xi )^\alpha -A) ^{-1} \tau ^{-\alpha }\delta (\xi )^\alpha \frac{\xi }{1-\xi } v, \end{aligned}$$

where the right-hand side is an analytic function in the unit disk. For $\rho \in (0,1)$, the Cauchy’s integral formula and the change of variable $\xi =e^{-\tau z}$ yield

$$\begin{aligned} Au^n&=\frac{1}{2\pi \mathrm {i}}\int _{|\xi |=\rho } Au(\xi ) \xi ^{-n-1} d\xi =\frac{\tau }{2\pi \mathrm i}\int _{\varGamma _\rho ^\tau } Au(e^{-\tau z}) e^{t_{n} z} d z =\frac{\tau }{2\pi \mathrm i}\int _{\varGamma _\rho ^\tau } K(z)v d z, \end{aligned}$$

where the kernel function *K*(*z*) is defined by

$$\begin{aligned} K(z)= e^{t_{n} z} A(\tau ^{-\alpha }\delta (e^{-\tau z})^\alpha -A) ^{-1}\tau ^{-\alpha }\delta (e^{-\tau z})^\alpha \frac{e^{-\tau z}}{1-e^{-\tau z}}, \end{aligned}$$

and $\varGamma _\rho ^\tau =\{ a+\mathrm{i} y: y\in (-\pi /\tau ,\pi /\tau )\}$ with $a=\tau ^{-1}\ln \frac{1}{\rho }>0$. Since *zR*(*z*; *A*) is bounded for $z\in \varSigma _{\alpha \pi /2}$, *zR*(*z*; *A*) is also bounded for $z\in \varSigma _{\alpha \pi /2+\varepsilon }$ (the angle can be slightly self-improved (cf. [42, Theorem 5.2 (c)]). Then a standard perturbation argument shows that there exists $\theta _\varepsilon >0$ (depending on $\varepsilon$) such that $\delta (e^{-\tau z})^\alpha \in \varSigma _{\alpha \pi /2+\varepsilon }$ when $z\in \varSigma _{\frac{\pi }{2}+\theta _\varepsilon }$. Let

$$\begin{aligned}&\varGamma _{\theta _\varepsilon ,\kappa }^\tau = \left\{ \rho e^{\mathrm{i}\theta _\varepsilon }: \kappa \le \rho \le \frac{\pi }{\tau \sin \theta _\varepsilon }\Big \} \bigcup \Big \{\kappa e^{\mathrm{i}\varphi }: -\theta _\varepsilon \le \varphi \le \theta _\varepsilon \right\} ,\\&\varGamma _{\pm }^\tau =\left\{ x \pm \mathrm{i} \pi /\tau : \frac{\pi \cos \theta _\varepsilon }{\tau \sin \theta _\varepsilon }<x< \tau ^{-1}\ln \frac{1}{\rho } \right\} , \end{aligned}$$

where $\varGamma _{\theta _\varepsilon ,\kappa }^\tau$ is oriented upwards and $\varGamma _\pm ^\tau$ is oriented rightwards, and $0<\kappa <\tau ^{-1}\ln \frac{1}{\rho }$. Then the function *K*(*z*)*v* is analytic in *z* in the region enclosed by $\varGamma _{\theta _\varepsilon ,\kappa }^\tau$, $\varGamma _{\pm }^\tau$ and $\varGamma _\rho ^\tau$. Since the integrals on $\varGamma _{+}^\tau$ and $\varGamma _{-}^\tau$ cancel each other due to the $2\pi \mathrm{i}$-periodicity of the integrand, the Cauchy’s theorem yields

$$\begin{aligned} Au^n = \frac{\tau }{2\pi \mathrm i}\int _{\varGamma _\rho ^\tau } K(z)v d z = \frac{\tau }{2\pi \mathrm i} \int _{\varGamma _{\theta _\varepsilon ,\kappa }^\tau } K(z)v d z. \end{aligned}$$

Then by choosing $\kappa =t_n^{-1}$ in the contour $\varGamma _{\theta _\varepsilon ,\kappa }^\tau$, we deduce

$$\begin{aligned} \Vert Au^n\Vert _X&\le c\left( \int _{\kappa t_n}^{\frac{\pi }{\tau \sin \theta _\varepsilon }} s^{-1} e^{s \cos \theta _\varepsilon } d s +\int _{-\theta _\varepsilon }^{\theta _\varepsilon } e^{t_{n} \kappa \cos \varphi }d \varphi \right) \Vert Av\Vert _X \le c\Vert Av\Vert _X. \end{aligned}$$

Similarly, one can show

$$\begin{aligned} \Vert u^n\Vert _X\le c\Vert v\Vert _X \quad \hbox {and}\quad \Vert Au^n\Vert _X\le ct_n^{-\alpha } \Vert v\Vert _X. \end{aligned}$$

This last estimate immediately implies the third assertion of Theorem 11. Now for $p\in (1/\alpha ,\infty ]$, we denote by $E_\tau :X\rightarrow L^\infty ({\mathbb R}_+,X)$ the operator which maps *v* to the piecewise constant function

$$\begin{aligned} E_\tau v= u_n\quad \forall \, t\in (t_{n-1},t_n], \quad n=1,2,\dots \end{aligned}$$

The preceding two estimates imply

7.6 $$\begin{aligned}&\Vert E_\tau v\Vert _{L^\infty ({\mathbb R}_+,D(A))}\le c\Vert v\Vert _{D(A)}, \end{aligned}$$

7.7 $$\begin{aligned}&\Vert E_\tau v\Vert _{L^{1/\alpha ,\infty }({\mathbb R}_+,D(A))}\le c \Vert v\Vert _X. \end{aligned}$$

The estimate (7.6) implies the first assertion of Theorem 11 in the case $p=\infty$, and the estimate (7.7) implies the second assertion of Theorem 11. The real interpolation of the last two estimates yields

$$\begin{aligned}&\Vert E_\tau v\Vert _{(L^{1/\alpha ,\infty }({\mathbb R}_+,D(A)),L^\infty ({\mathbb R}_+,D(A)))_{1-\frac{1}{\alpha p},p}} \le c\Vert v\Vert _{(X,D(A))_{1-\frac{1}{\alpha p},p}}, \,\,\, \forall \, p \in (\alpha ^{-1}, \infty ). \end{aligned}$$

Since $(L^{1/\alpha ,\infty }({\mathbb R}_+,D(A)),L^\infty ({\mathbb R}_+,D(A)))_{1-\frac{1}{\alpha p},p}=L^p({\mathbb R}_+,D(A))$ [9, Theorem 5.2.1], this implies the first assertion of Theorem 11 in the case $p\in (1/\alpha ,\infty )$. $\square$

### Remark 6

The proof shows that in the absence of a source term *f*, the maximal $\ell ^p$-regularity of (7.3) only requires the sectorial property of *A*, rather than the *R*-sectorial property. The general case (with nonzero source and nonzero initial data) is a linear combination of (1.3) and (7.2).

### Remark 7

We have focused our discussions on the Caputo fractional derivative, since it allows specifying the initial condition as usual, and thus is very popular among practitioners. In the Riemann–Liouville case, generally it requires integral type initial condition(s) [27], for which the physical interpretation seems unclear.

In the proof of Theorem 11, we first prove two end-point cases, $p=1/\alpha$ and $p=\infty$. Then we use real interpolation method for the case $1/\alpha<p<\infty$. The real interpolation method also holds for $0<p<1$ ([9, Theorem 5.2.1]). Thus, we have the following theorem in the case $1<\alpha <2$. The proof is omitted, since it is almost identical with the proof of Theorem 11.

### Theorem 12

Let *X* be a Banach space, $1<\alpha <2$, and let *A* be a sectorial operator on *X* of angle $\alpha \pi /2$. Then the BE scheme (7.3) has the following maximal $\ell ^p$-regularity:

$$\begin{aligned}&\Vert (\bar{\partial }_\tau ^\alpha u^n)_{n=1}^N\Vert _{L^p(X)}+ \Vert (Au^n)_{n=1}^N\Vert _{L^p(X)} \\&\le \left\{ \begin{array}{l@{\quad }l} c_{p} (\Vert v\Vert _{(X,D(A))_{1-\frac{1}{p\alpha },p}}+\Vert w\Vert _{X}), &{}\hbox {for}\,\,\, p\in \Big [1,\frac{1}{\alpha -1}\Big ), \\ c_{p} (\Vert v\Vert _{(X,D(A))_{1-\frac{1}{p\alpha },p}}+\Vert w\Vert _{(X,D(A))_{1-\frac{1}{\alpha }-\frac{1}{p\alpha },p}}), &{}\hbox {for}\,\,\, p\in \Big (\frac{1}{\alpha -1},\infty \Big ], \end{array} \right. \end{aligned}$$

and

$$\begin{aligned} \Vert (\bar{\partial }_\tau ^\alpha u^n)_{n=1}^N\Vert _{L^{p,\infty }(X)}&+ \Vert (Au^n)_{n=1}^N\Vert _{L^{p,\infty }(X)} \\&\le c_{p} (\Vert v\Vert _{(X,D(A))_{1-\frac{1}{p\alpha },p}}+\Vert w\Vert _{X}),&\hbox {for}\,\,\, p=\frac{1}{\alpha -1}, \end{aligned}$$

where the constant $c_{p}$ depends on the bound of the set of operators $\{zR(z;A) :z\in \varSigma _{\alpha \pi /2}\}$, independent of *N*, $\tau$ and *A*.

## Examples and application to error estimates

In this section, we present a few examples of fractional evolution equations which possess the maximal $L^p$-regularity, and investigate conditions under which the time-stepping schemes in Sects. 3–6 satisfy the maximal $\ell ^p$-regularity.

### Example 1

(**Continuous problem**) Consider the following time fractional parabolic equation in a bounded smooth domain $\varOmega \subset {\mathbb R}^d$ ($d\ge 1$):

8.1 $$\begin{aligned} \left\{ \begin{array}{l@{\quad }l} \partial _t^\alpha u(x,t) = \varDelta u(x,t) + f(x,t) &{}\hbox {for}\,\,\,(x,t)\in \varOmega \times (0,T),\\ u(x,t) = 0 &{}\hbox {for}\,\,\,(x,t)\in \partial \varOmega \times (0,T),\\ u(x,0) = 0&{}\hbox {for}\,\,\,x\in \varOmega ,\quad \hbox {if }0<\alpha<1,\\ u(x,0) =\partial _t u(x,0) = 0 &{}\hbox {for}\,\,\,x\in \varOmega ,\quad \hbox {if } 1<\alpha <2, \end{array} \right. \end{aligned}$$

where $T>0$ is given and $\varDelta$ denotes the Laplacian operator. In the appendix, we show that the $L^q$ realization $\varDelta _q$ in $X=L^q(\varOmega )$ of $\varDelta$ is an *R*-sectorial operator in *X* with angle $\theta \in (0,\pi )$, and that $\varDelta _q v$ coincides with $\varDelta v$ in the domain $D(\varDelta _q)$ of $\varDelta _q$:

8.2 $$\begin{aligned} \varDelta _q v=\varDelta v, \quad \forall \, v\in D(\varDelta _q),\quad \forall \, 1<q<\infty . \end{aligned}$$

Thus Theorem 3 implies that the solution $u_q$ of

8.3 $$\begin{aligned} \left\{ \begin{aligned}&\partial _t^\alpha u_q = \varDelta _q u_q + f,\\&u_q(\cdot ,0) = 0&\hbox {if}\,\,\,0<\alpha<1,\\&u_q(\cdot ,0) =\partial _t u_q(\cdot ,0) = 0&\hbox {if}\,\,\,1<\alpha <2, \end{aligned} \right. \end{aligned}$$

satisfies $u_q(\cdot ,t)\in D(\varDelta _q)$ for almost all $t \in \mathbb {R}_+$ and

8.4 $$\begin{aligned} \begin{aligned}&\quad \Vert \partial _t^\alpha u_q \Vert _{L^p(0,T;L^q(\varOmega ))} + \Vert \varDelta _q u_q\Vert _{L^p(0,T;L^q(\varOmega ))} \\&\le \Vert \partial _t^\alpha u_q \Vert _{L^p({\mathbb R}_+;L^q(\varOmega ))} + \Vert \varDelta _q u_q\Vert _{L^p({\mathbb R}_+;L^q(\varOmega ))} \\&\le c_{p,X}\Vert f\Vert _{L^p({\mathbb R}_+;L^q(\varOmega ))} \\&= c_{p,X}\Vert f\Vert _{L^p(0,T;L^q(\varOmega ))},\qquad \forall \, 1<p,q<\infty . \end{aligned} \end{aligned}$$

In view of (8.2), we shall denote $(\varDelta _q,D(\varDelta _q))$ by $(\varDelta ,D_q(\varDelta ))$ below. Then (8.2)–(8.4) imply that for any given $1<p,q<\infty$ and $f\in L^p(0,T;L^q(\varOmega ))$, problem (8.1) has a unique solution $u\in L^p(0,T;D_q(\varDelta ))\cap W^{1,p}(0,T;L^q(\varOmega ))$ satisfying the maximal regularity

$$\begin{aligned} \Vert \partial _t^\alpha u \Vert _{L^p(0,T;L^q(\varOmega ))} + \Vert \varDelta u\Vert _{L^p(0,T;L^q(\varOmega ))} \le c_{p,X}\Vert f\Vert _{L^p(0,T;L^q(\varOmega ))}. \end{aligned}$$

### Example 2

(**Time discretization**) Since the Dirichlet Laplacian $\varDelta :D_q(\varDelta )\rightarrow L^q(\varOmega )$ defined in Example 8.1 is *R*-sectorial of angle $\theta$ for all $\theta \in (0,\pi )$, Theorems 5, 6 and 7 imply that the time (semi-)discrete solutions given by the backward Euler, BDF2 and L1 scheme satisfy the following maximal $\ell ^p$-regularity:

8.5 $$\begin{aligned} \Vert (\bar{\partial }_\tau ^\alpha u^n)_{n=1}^N\Vert _{\ell ^p(L^q(\varOmega ))}+ \Vert (\varDelta u^n)_{n=1}^N\Vert _{\ell ^p(L^q(\varOmega ))}\le c_{p,q}\Vert (f^n)_{n=0}^N\Vert _{\ell ^p(L^q(\varOmega ))}. \end{aligned}$$

By Theorem 9, the fractional Crank–Nicolson solution also satisfies (8.5) when $0<\alpha <1$. Since $\varDelta$ is self-adjoint and has an unbounded spectrum, it follows that $r(\varDelta )=\infty$, so the conditions of Theorems 8 and 10 cannot be satisfied.

### Example 3

(**Space–time fractional PDE**) Consider the following space–time nonlocal parabolic equation in ${\mathbb R}^d$ ($d\ge 1$):

8.6 $$\begin{aligned} \left\{ \begin{array}{l@{\quad }l} \partial _t^\alpha u(x,t) =-(-\varDelta )^\frac{1}{2} u(x,t) + f(x,t) &{}\hbox {for}\,\,\,(x,t)\in {\mathbb R}^d\times {\mathbb R}^+,\\ u(x,0) = 0 &{}\hbox {for}\,\,\,x\in {\mathbb R}^d,\quad \hbox {if}\,\,\,0<\alpha<1,\\ u(x,0) =\partial _t u(x,0) = 0 &{}\hbox {for}\,\,\,x\in {\mathbb R}^d,\quad \hbox {if}\,\,\,1<\alpha <2, \end{array} \right. \end{aligned}$$

where the fractional Laplacian $(-\varDelta )^\frac{1}{2}v$ is defined by

$$\begin{aligned} (-\varDelta )^\frac{1}{2}v:={\mathcal F}^{-1}_\xi \big (|\xi |({\mathcal F}v)(\xi )\big ), \quad \forall \, v\in W^{1,q}({\mathbb R}^d). \end{aligned}$$

For $X:=L^q({\mathbb R}^d)$ and $D_q((-\varDelta )^\frac{1}{2} ) := W^{1,q}({\mathbb R}^d)$, $1< q<\infty$, the fractional operator $-(-\varDelta )^\frac{1}{2}:W^{1,q}({\mathbb R}^d)\rightarrow L^q({\mathbb R}^d)$ is also *R*-sectorial of angle $\theta$ for arbitrary $\theta \in (0,\pi )$ [1, proof of Proposition 2.2]. Hence, by Theorems 5, 6 and 7, the backward Euler, BDF2 and L1 schemes all satisfy the following maximal $\ell ^p$-regularity when $0<\alpha <2$ and $\alpha \ne 1$

$$\begin{aligned} \Vert (\bar{\partial }_\tau ^\alpha u^n)_{n=1}^N\Vert _{\ell ^p(L^q(\varOmega ))}+ \Vert ((-\varDelta )^\frac{1}{2} u^n)_{n=1}^N\Vert _{\ell ^p(L^q(\varOmega ))}\le c_{p,q}\Vert (f^n)_{n=0}^N\Vert _{\ell ^p(L^q(\varOmega ))}. \end{aligned}$$

By Theorem 9, the fractional Crank–Nicolson scheme also satisfies this estimate when $0<\alpha <1$.

### Example 4

(**Fractional PDEs with complex coefficients**) Consider the following time-fractional PDE with a complex coefficient in a bounded Lipschitz domain $\varOmega \subset {\mathbb R}^d$ ($d\ge 1$):

8.7 $$\begin{aligned} \left\{ \begin{array}{l@{\quad }l} \partial _t^\alpha u(x,t) -e^{\mathrm{i}\varphi }\varDelta u(x,t) =f(x,t) &{}\hbox {for}\,\,\,(x,t)\in \varOmega \times {\mathbb R}^+,\\ u (x,t) = 0 &{} \hbox {for}\,\,\, (x,t)\in \partial \varOmega \times \mathbb {R}^+,\\ u(x,0) = 0 &{}\hbox {for}\,\,\,x\in \varOmega ,\quad \hbox {if}\,\,\,0<\alpha<1,\\ u(x,0) =\partial _t u(x,0) = 0 &{}\hbox {for}\,\,\,x\in \varOmega ,\quad \hbox {if}\,\,\,1<\alpha <2, \end{array} \right. \end{aligned}$$

where $\varphi \in (-\pi ,\pi )$ is given. It is worth noting that if $\varphi \in (\pi /2,\pi )\cup (-\pi ,-\pi /2)$, then (8.7) is a diffusion-wave problem, since the operator $-e^{\mathrm{i}\varphi }\varDelta$ has eigenvalues with negative real part. For $X:=L^q(\varOmega )$ and $D_q(e^{\mathrm{i}\varphi }\varDelta ):=D_q(\varDelta )$, $1< q<\infty$, the operator $e^{\mathrm{i}\varphi }\varDelta :D_q(\varDelta )\rightarrow L^q(\varOmega )$ is *R*-sectorial of angle $\theta$ for arbitrary $\theta \in (0,\pi -\varphi )$. Hence, by Theorems 5, 6 and 7, the backward Euler, BDF2 and L1 schemes satisfy the maximal $\ell ^p$-regularity estimate (8.5) when $0<\alpha <2-2\varphi /\pi$ and $\alpha \ne 1$; the fractional Crank–Nicolson scheme also satisfies the estimate (8.5) when $0<\alpha <\min (2-2\varphi /\pi ,1)$.

As an application of the maximal $\ell ^p$-regularity, we present error estimates for the numerical solutions by the BE scheme (3.1), with the scaled $L^p$-norm (7.4). Other time-stepping schemes can be analyzed similarly.

### Theorem 13

Let $A:D(A)\rightarrow X$ be an *R*-sectorial operator of angle $\alpha \pi /2$, with $\alpha \in (0,2)$ and $\alpha \ne 1$, and the solution *u* of (1.3) be sufficiently smooth. Then the solution of the BE scheme (3.1) satisfies for any $1<p<\infty$

8.8 $$\begin{aligned} \Vert \bar{\partial }_\tau ^\alpha (u^n- u(t_n))_{n=1}^N \Vert _{L^p(X)} + \Vert A(u^n-u(t_n))_{n=1}^N\Vert _{L^p(X)} \le c_{p}\, \tau . \end{aligned}$$

### Proof

We denote by $e^n:=u^n-u(t_n)$ the error of the numerical solution $u^n$. Then $e^n$ satisfies

8.9 $$\begin{aligned} \bar{\partial }_\tau ^\alpha e^n = A e^n -E^n, \quad n=1,2,\dots \end{aligned}$$

with $e^0=0$, where $E^n:=\bar{\partial }_\tau ^\alpha u(t_n)-\partial _t^\alpha u(t_n)$ denotes the truncation error, satisfying $\displaystyle \max _{1\le n\le N}\Vert E^n\Vert _{X} \le c\tau$ [47]. By applying Theorem 5 to (8.9), we obtain

$$\begin{aligned} \Vert (\bar{\partial }_\tau ^\alpha e^n)_{n=1}^N\Vert _{L^p(X)} + \Vert (Ae^n)_{n=1}^N\Vert _{L^p(X)}\le c_{p,X}c_R\Vert (E^n)_{n=1}^N\Vert _{L^p(X)} \le c_p\, \tau . \end{aligned}$$

$$\square $$

If $\varOmega$ is a bounded smooth domain, $X=L^q(\varOmega )$, $1<q<\infty$, $D(A)=W^{2,q}(\varOmega )\cap W^{1,q}_0(\varOmega )$ and $A=\varDelta$ (the Dirichlet Laplacian), then the conditions of Theorem 13 are satisfied, provided that the solution *u* is smooth, and (8.8) gives that for any $1<p,q<\infty$

$$\begin{aligned} \Vert (u^n-u(t_n))_{n=1}^N\Vert _{L^p(W^{2,q}(\varOmega ))} \le c_{q}\Vert \varDelta (u^n-u(t_n))_{n=1}^N\Vert _{L^p(L^q(\varOmega ))} \le c_{p,q}\, \tau . \end{aligned}$$

When $q>d$, error estimates in such strong norms as $W^{2,q}(\varOmega )\hookrightarrow W^{1,\infty }(\varOmega )$ can be used to control some strong nonlinear terms in the numerical analysis of nonlinear parabolic problems [1, 2, 17]. We will explore such an analysis in the future.

## Appendix: *R*-sectorial property of $\varDelta _q$

In this appendix, we show that the $L^q$ realization $\varDelta _q$ in $X=L^q(\varOmega )$ of $\varDelta$ is an *R*-sectorial operator in *X* with angle $\theta \in (0,\pi )$ (see also [2, Lemma 8.2] for related discussions).

Let $\varDelta _2$ be the restriction of the operator $\varDelta$ to the domain $D(\varDelta _2)=\{v\in H^1_0(\varOmega ):\varDelta v\in L^2(\varOmega )\}$. Then the densely defined self-adjoint operator $\varDelta _2: D(\varDelta _2)\rightarrow L^2(\varOmega )$ generates a bounded analytic semigroup $E_2(t):L^2(\varOmega )\rightarrow L^2(\varOmega )$ [3, Example 3.7.5], which extends to a bounded analytic semigroup $E_q(t)$ on $L^q(\varOmega )$, $1<q<\infty$ [41, Theorem 3.1], such that

A.1 $$\begin{aligned}&E_{q_1}(t)v=E_{q_2}(t)v,&\forall \, v\in L^{q_1}(\varOmega )\cap L^{q_2}(\varOmega ), \nonumber \\&E_q(t)v=\int _\varOmega G(t,x,y)v(y) dy,&\forall \, v\in L^q(\varOmega ), \end{aligned}$$

where *G*(*t*, *x*, *y*) is the kernel of the semigroup $E_2(t)$, i.e., the parabolic Green’s function. It satisfies the following Gaussian estimate [12, Corollary 3.2.8]:

A.2 $$\begin{aligned} 0 \le G(t,x,y) \le ct^{-\frac{d}{2}}e^{-\frac{|x-y|^2}{ct}}. \end{aligned}$$

Let $\varDelta _q$ be the generator of the semigroup $E_q(t)$, with its domain [3, Proposition 3.1.9, g]

A.3 $$\begin{aligned} D(\varDelta _q)=\left\{ v\in L^q(\varOmega ): \lim _{t\downarrow 0}\frac{E_q(t)v-v}{t}\,\,\hbox {exists in }L^q(\varOmega )\right\} . \end{aligned}$$

(A.1) and (A.3) imply that

$$\begin{aligned}&D(\varDelta _{q_2})\subset D(\varDelta _{q_1})&\hbox {for}\; 1<q_1<q_2<\infty ,\\&\varDelta _{q_1}v=\varDelta _{q_2}v&\hbox {for }\; v\in D(\varDelta _{q_2})\cap D(\varDelta _{q_1}). \end{aligned}$$

In particular, we have

A.4 $$\begin{aligned} \varDelta _qv=\varDelta v,\quad \forall \, v\in D(\varDelta _q)\cap D(\varDelta _2), \quad \forall \, 1<q<\infty . \end{aligned}$$

The Gaussian estimate (A.2) yields

$$\begin{aligned} \Vert E_q(t/2)v\Vert _{L^2(\varOmega )}\le ct^{-\frac{d}{2}}\Vert v\Vert _{L^1(\varOmega )} \le ct^{-\frac{d}{2}}\Vert v\Vert _{L^q(\varOmega )},\quad \forall \, v\in L^q(\varOmega ). \end{aligned}$$

That is, $E_q(t/2)v\in L^2(\varOmega )$ for $v\in L^q(\varOmega )$ and $t>0$. Hence, (A.1) implies

A.5 $$\begin{aligned} E_q(t)v=E_q(t/2)E_q(t/2)v=E_2(t/2)E_q(t/2)v\in D(\varDelta _2), \end{aligned}$$

where the last inclusion is due to the analyticity of the semigroup $E_2(t)$ [3, Theorem 3.7.19]. Then (A.4) and (A.5) imply

$$\begin{aligned} \lim _{t\downarrow 0}\Vert \varDelta E_q(t) v-\varDelta _q v\Vert _{L^q(\varOmega )}&= \lim _{t\downarrow 0} \Vert \varDelta _q E_q(t) v-\varDelta _q v\Vert _{L^q(\varOmega )} = 0, \quad \forall \, v\in D(\varDelta _q). \end{aligned}$$

Since $\displaystyle \lim _{t\downarrow 0}\Vert E_q(t) v-v\Vert _{L^q(\varOmega )}=0$, the last identity implies

$$\begin{aligned} (\varDelta _q v,\varphi )&= \lim _{t\downarrow 0}(\varDelta E_q(t) v,\varphi ) =\lim _{t\downarrow 0}(E_q(t) v,\varDelta \varphi ) =(v,\varDelta \varphi ),\\&\qquad \forall \, v\in D(\varDelta _q),\,\,\,\forall \,\varphi \in C^\infty _0(\varOmega ). \end{aligned}$$

That is, $\varDelta _q v$ coincides with the distributional partial derivative $\varDelta v$ in the sense of distributions, i.e.,

A.6 $$\begin{aligned} \varDelta _q v=\varDelta v, \quad \forall \, v\in D(\varDelta _q),\quad \forall \, 1<q<\infty . \end{aligned}$$

### Remark 8

If the domain $\varOmega$ is smooth or convex, then we have the characterization

$$\begin{aligned} D(\varDelta _q)=\{v\in W^{1,q}_0(\varOmega ): \varDelta v\in L^q(\varOmega )\}. \end{aligned}$$

However, this characterization does not hold in general bounded Lipschitz domains (e.g., nonconvex polygons). In a general bounded Lipschitz domain, the operator $\varDelta _2^{-1}:L^2(\varOmega )\rightarrow L^2(\varOmega )$ has an extension $\varDelta ^{-1}:L^1(\varOmega )\rightarrow L^1(\varOmega )$, given by [18]

$$\begin{aligned} \varDelta ^{-1}v(x)=\int _\varOmega {\mathcal G}(x,y)v(y) dy \end{aligned}$$

in terms of the elliptic Green’s function ${\mathcal G}(x,y)$, satisfying

$$\begin{aligned}&\varDelta ^{-1}v=\varDelta _q^{-1}v, \quad \forall \, v\in L^q(\varOmega ). \end{aligned}$$

Hence, we have the characterization $D(\varDelta _q)=\{\varDelta ^{-1}v:v\in L^q(\varOmega )\}$.
